# Implications of regulatory T cells in non-lymphoid tissue physiology and pathophysiology

**DOI:** 10.3389/fimmu.2022.954798

**Published:** 2022-07-22

**Authors:** Darya Malko, Tarek Elmzzahi, Marc Beyer

**Affiliations:** ^1^ Department of Microbiology and Immunology, The Peter Doherty Institute for Infection and Immunity, University of Melbourne, Melbourne, VIC, Australia; ^2^ Immunogenomics and Neurodegeneration, Deutsches Zentrum für Neurodegenerative Erkrankungen (DZNE), Bonn, Germany; ^3^ Platform foR SinglE Cell GenomIcS and Epigenomics (PRECISE), Deutsches Zentrum für Neurodegenerative Erkrankungen (DZNE) and University of Bonn, Bonn, Germany

**Keywords:** regulatory T cells, Treg cells, tissue homeostasis, FoxP3, tissue Treg cells, nonlymphoid tissues, tissue repair

## Abstract

Treg cells have been initially described as gatekeepers for the control of autoimmunity, as they can actively suppress the activity of other immune cells. However, their role goes beyond this as Treg cells further control immune responses during infections and tumor development. Furthermore, Treg cells can acquire additional properties for e.g., the control of tissue homeostasis. This is instructed by a specific differentiation program and the acquisition of effector properties unique to Treg cells in non-lymphoid tissues. These tissue Treg cells can further adapt to their tissue environment and acquire distinct functional properties through specific transcription factors activated by a combination of tissue derived factors, including tissue-specific antigens and cytokines. In this review, we will focus on recent findings extending our current understanding of the role and differentiation of these tissue Treg cells. As such we will highlight the importance of tissue Treg cells for tissue maintenance, regeneration, and repair in adipose tissue, muscle, CNS, liver, kidney, reproductive organs, and the lung.

## Introduction

**Figure 1:**
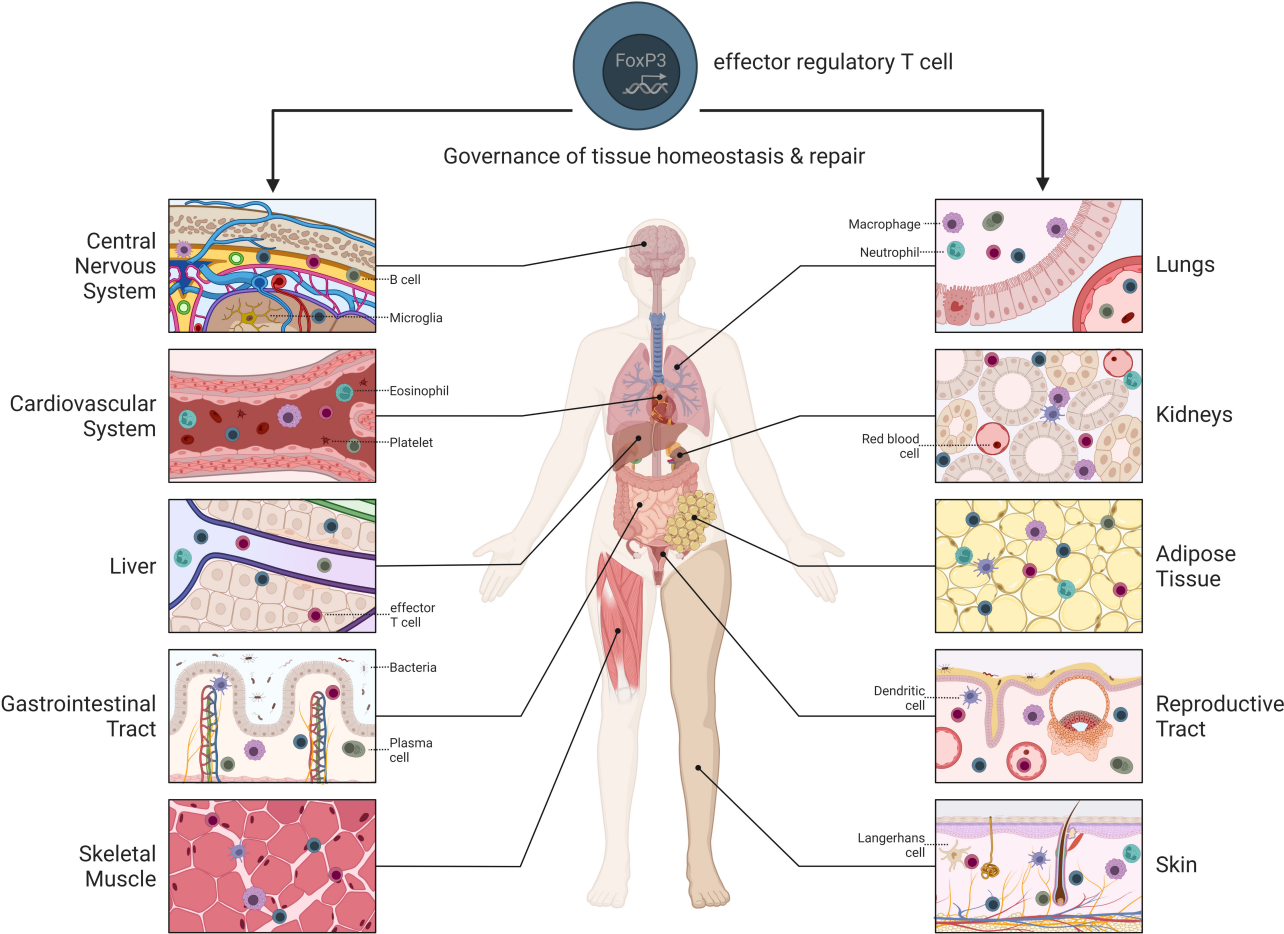
Overview of tissue-specific Treg cells throughout the body. Treg cells reside within different non-lymphoid tissues where they contribute to tissue maintenance and repair.

Regulatory T cells (Treg cells) are a specialized subpopulation of the CD4^+^ T cell lineage which play an indispensable role in maintaining immune homeostasis, inducing peripheral tolerance, and controlling inflammation (1). While initial work focused on the identification of general functional properties of Treg cells, in the past years, distinct effector Treg cell populations within non-lymphoid organs have been described (2) (**Figure 1**). Treg cells in the periphery can adopt specialized differentiation programs resulting in the acquisition of tissue-specific phenotypes, propelling tissue-specific Treg cell functionality. This goes along with the development of a transcriptional effector Treg cell program. As we describe below, this program has commonalities across different tissues, but also has peculiarities specific for each tissue. These tissue-specific properties are often instructed by the respective tissue and as such have been described as being hijacked by Treg cells to adapt to the tissue microenvironment.

While we acknowledge the existence of CD8^+^ Treg cells in mice and humans which might also be indispensable within tissues (3–6), this review will focus on CD4^+^ Treg cells and further elucidate the important role of Treg cells in non-lymphoid tissue physiology and pathophysiology while largely excluding their contribution to the control of autoimmunity, infections, and tumorigenesis.

### Development of Treg cells

T cell progenitors develop in the bone marrow from multipotent hematopoietic stem cells and migrate to the thymus where rearrangement of the T cell receptor (TCR) genes occurs. Subsequently, thymic Treg cell development takes place. The classical model for Treg cell development in the thymus comprises two steps: intermediate-strength TCR signaling that promotes surface expression of the interleukin (IL)-2 receptor subunit CD25, followed by IL-2/IL-15 signaling that induces expression of the lineage-defining transcription factor forkhead box P3 (FoxP3) (7). FoxP3 subsequently orchestrates the epigenetic and transcriptional landscape required for the acquisition of the classical Treg cells phenotype. An alternative scenario involves the induction of low expression of FoxP3 upon TCR stimulation, followed by similar cytokine signaling to complete Treg cell differentiation (7). Independent of the exact molecular pathway, thymus-derived Treg cell development further depends on the avidity and affinity of the molecular interactions of the TCR with antigen within the major histocompatibility complex (MHC) requiring a higher affinity to self-antigens then CD25-negative T cells (8–11). Moreover, co-stimulation *via* the CD28 - CD80/CD86 axis promotes the expression of appropriate FoxP3 levels, necessary for the subsequent upregulation of the Treg cell-associated gene expression program including upregulation of cell-surface molecules like cytotoxic T lymphocyte antigen 4 (CTLA-4) and glucocorticoid-induced tumor necrosis factor receptor family-related receptor (GITR) (12, 13).

Besides thymic Treg cells, a second population of Treg cells with specific properties can be generated in the periphery. These peripherally induced Treg cells develop from naïve CD4^+^ T cells upon antigen stimulation in the presence of cytokines such as transforming growth factor beta (TGF-β) and IL-2 but require absence of pro-inflammatory cytokines (14–17). Particularly, TGF-β promotes the transcription of FoxP3 (18, 19) with factors such as retinoic acid further supporting the induction of FoxP3 expression and its stabilization through demethylation of enhancer elements at the FoxP3 locus. IL-2 facilitates this TGF-β-mediated differentiation of Treg cells by directly promoting Treg cell survival and expansion (20–22) and inhibiting T helper (Th)17 cell development at the same time (23, 24). While TGF-β-mediated T cell differentiation mainly leads to Treg cell development under homeostatic conditions, pro-inflammatory conditions characterized by the additional presence of e.g., IL-6 favor Th17 over Treg cell differentiation (17, 25, 26).

### Naïve versus effector Treg cells

**Figure 2:**
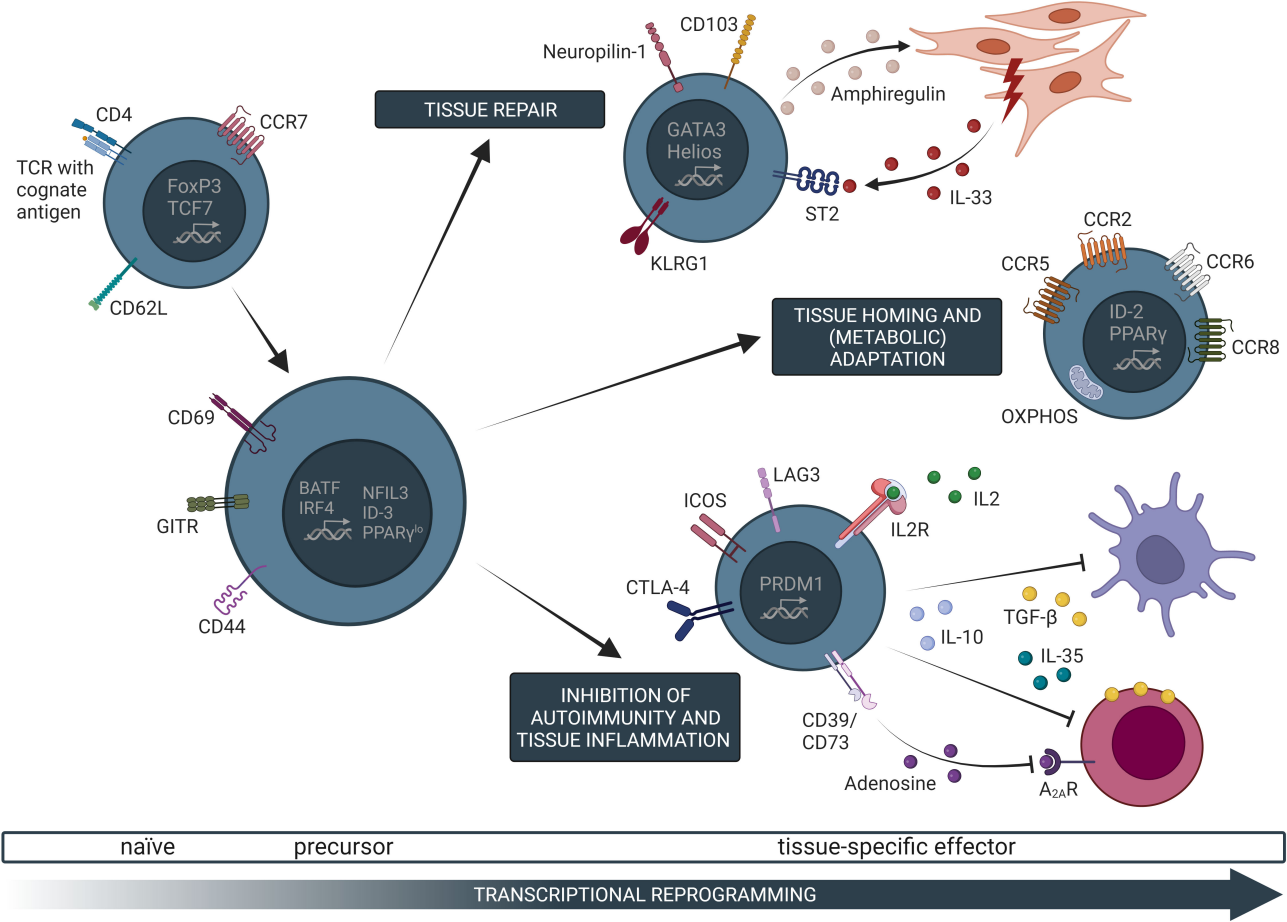
Common transcriptional, phenotypic, and functional characteristics of tissue-specific Treg cells. Naive Treg cells in secondary lymphoid organs can undergo T cell receptor (TCR)-dependent activation, which initiates a gradual transcriptional programming trajectory. Specifically, activated Treg cells downregulate canonical markers of naive T cells, such as CD62L, and upregulate expression of genes related to T cell activation (e.g. CD69, CD44), self-renewal (e.g. ID-3), as well as typical non-lymphoid tissue Treg cell marker (e.g. PPARγ). Such Treg cell precursors possess the capacity to further differentiate into tissue-specific Treg cells. These cells migrate into non-lymphoid tissues along chemokine gradients (e.g. CCL2/CCR2 for the VAT) where they locally control immune and tissue homeostasis. Once in the tissue, Treg cells further undergo transcriptional as well as metabolic adaptations to the assigned tissue microenvironment, resulting in a tissue-specific effector phenotype. The contribution of effector Treg (eTreg) cells to tissue regeneration and repair has been identified as a common characteristic in various non-lymphoid tissues such as VAT, muscle, or the kidneys where this effect is mainly dependent on IL-33 and amphiregulin. Furthermore, common eTreg cell modules (e.g. KLRG1, ICOS, or CTLA-4) are shared between different tissues. Controlling inflammation is one of the main functions of Treg cells and has also been described in tissue-settings by mechanisms such as the release of suppressive cytokine or metabolic disruption of the target cells. OXPHOS, oxidative phosphorylation; TCF7, transcription factor 7.

In analogy to conventional CD4^+^ T cells (Tconv cells), Treg cells exiting the thymus display a naïve phenotype, expressing lymphoid tissue-homing receptors such as L-selectin and C-C chemokine receptor type 7 (CCR7). Upon encountering their cognate (self-)antigen, TCR stimulation induces transcriptional changes that drive naïve-like thymus-derived Treg cells circulating in secondary lymphoid organs to differentiate into effector Treg cells (eTreg cells) (27) (**Figure 2**). Such eTreg cells possess high proliferation rates and superior suppressive functions, manifested by increased expression of surface markers such as Killer Cell Lectin Like Receptor G1 (KLRG1) and CTLA-4, and enhanced release of IL-10 (27). Furthermore, these eTreg cells gain the function to migrate into peripheral tissues and locally control immune and tissue homeostasis.

Over the last years, increasing evidence supports a hierarchical program of transcription factors governing this differentiation process. Besides FoxP3, expression of the transcription factors PR domain zinc finger protein 1 (PRDM1, also known as Blimp-1) and interferon regulatory factor 4 (IRF4) have been identified as key steps for the differentiation, function, and homeostasis of eTreg cells (28). Expression of both transcription factors results in high expressions of CD103, CD44, inducible costimulator (ICOS), GITR, CD38, and CD69, as well as IL-10 production consistent with an eTreg cell phenotype (28). This more general eTreg cell program is complemented through tissue specific adaptations as outlined below.

### Tissue Treg cell precursors in secondary lymphoid organs

A critical question concerning eTreg cells in non-lymphoid tissues involves their developmental origin. Specifically, studies explored whether tissue Treg cell precursors egress from the thymus in a state that enables them to directly home to their tissue destination, or whether they undergo gradual maturation in secondary lymphoid organs (SLOs) before completing their adaptation in the tissue of residence.

Under steady-state conditions, T cells do not exclusively exhibit a naïve state; rather, antigen-experienced T cells are still generated without overt perturbation of homeostasis (29). Similarly, Treg cells in SLOs do not uniformly display a naïve phenotype. Yang et al. (30) reported that Treg cells in spleen and peripheral lymph nodes are classified into three subsets based on the expression of CD62L and T cell factor-1 (TCF-1), which promote the longevity and self-renewal of naïve and memory T cells. The TCF-1^+^CD62L^+^ subset represented 45-60% of lymphoid-tissue Treg cells, and expressed markers of naïve, quiescent T cells (e.g., Myb, Satb1). Conversely, TCF-1^-^ CD62L^-^ Treg cells, representing <20% of the lymphoid-tissue Treg cell pool, were characterized by an eTreg cell phenotype, with i.e., increased expression of *Icos, Ctla4, Il10*, among other genes (30). These findings suggest that naïve Treg cells are instructed in lymphoid tissues to gradually adopt an eTreg cell program before their migration to non-lymphoid tissues. Further supporting this notion is a study by Li et al. identifying a small subset of splenic Treg cells expressing low levels of *Pparg* (31). Indeed, *Pparg* encodes peroxisome proliferator-activated receptor gamma (PPARγ), a critical transcription factor for adipocyte differentiation as well as for the generation and function of Treg cells in visceral adipose tissue (VAT) (32). This PPARγ-low Treg cell subset displayed a more activated phenotype compared to their PPARγ-negative counterparts in the spleen (31). Importantly, PPARγ-low Treg cells exhibited an enhanced capacity to give rise to mature tissue Treg cells compared to splenic PPARγ-negative Treg cells (31). Further characterization of splenic PPARγ-low Treg cell precursors revealed that they comprise two subpopulations with distinct transcriptional landscapes (33). Specifically, one of the precursor subsets expressed higher amounts of transcripts encoding chemokine receptors and integrins, whereas the second precursor subset upregulated the expression of Treg cell maturation and activation markers such as *Klrg1* and *Pdcd1*, among others. Moreover, clonal overlap between both splenic PPARγ-low Treg cells and Treg cells resident in adipose tissue, skin, and liver, suggested that such splenic Treg cells give rise to at least a fraction of the respective pools of tissue Treg cells (33). In summary, these data support a model, where PPARγ-negative splenic Treg cells gradually develop into PPARγ-low tissue Treg cell precursors, which in turn eventually differentiate into tissue Treg cells. (**Figure 2**).

Consistent with the notion of a common PPARγ-expressing precursor in the spleen, Delacher et al. found that chromatin at the genomic *Pparg* locus remains accessible in several non-lymphoid tissue Treg cells (34). Such observations of chromatin accessibility at the genomic *Pparg* locus hinted at shared epigenetic reprogramming events preceding the specification of the different tissue Treg cells. Based on the expression of KLRG1 and the transcription factor nuclear factor interleukin 3 (NFIL3), the authors subdivided splenic Treg cells into largely naïve KLRG1^-^NFIL3^-^Treg cells, and two subpopulations of Treg cell precursors. The population of NFIL3^+^ KLRG1- splenic precursors first needed to upregulate KLRG1 before subsequently giving rise to at least a fraction of the non-lymphoid tissue Treg cells in a basic leucine zipper transcription factor (BATF)-dependent fashion (34). In a follow-up study the same group confirmed these findings using single-cell chromatin accessibility data moreover showing a similar Treg cell signature in humans (35). In addition to BATF, CCR8 was identified as a marker for tissue-like Treg cells in human peripheral blood which, based on TCR-sequencing and pseudotime-based developmental trajectory analyses, were proposed as circulating Treg cell precursors for tissue-resident Treg cells in human fat and skin (35).

In addition, Sullivan et al. demonstrated that low expression of the transcription factor inhibitor of DNA binding (ID)-3 marks tissue Treg cell precursors in SLOs (36). Splenic Treg cells progressively downregulated ID-3 and upregulated ID-2 as they transitioned from a naïve state to an increasingly mature effector phenotype. In line with this observation, tissue Treg cells were found to express low levels of ID-3 (36).

Taken together, recent evidence supports that the transition from a naive phenotype to the full tissue Treg cell program is initiated already in SLOs, yet the differentiation and full adaptation to the tissue microenvironment only occurs once Treg cells have migrated into their assigned tissue (**Figure 2**).

## Specific properties of tissue Treg cells in adipose tissue

One of the best studied tissues to understand the events necessary for this instruction of tissue properties in Treg cells and their role for the maintenance of homeostasis in a non-lymphoid tissue is the adipose tissue. The adipose tissue can be classified into white adipose tissue (WAT), where fatty acids are deposited, and brown adipose tissue, which is more prone to thermogenesis (37). WAT is found either subcutaneously or in the abdominal viscera (37). Apart from being an energy store, VAT is characterized by a chronic, low-grade inflammation, particularly in male mice (38). Such VAT inflammation is exacerbated in the context of obesity, where it drives insulin resistance and glucose intolerance (39). Indeed, an increased VAT volume is a strong correlate of different parameters of metabolic disorders (40). VAT inflammation itself is mediated by different subsets of myeloid cells and Th1 effector T cells as well as adipocytes, releasing an array of proinflammatory cytokines and effector molecules (41, 42).

The physiologic mechanism through which mice limit inflammation in the VAT is exerted by a special population of tissue Treg cells residing in the VAT (39). Such VAT Treg cells modulate the inflammatory milieu as well as insulin sensitivity and, hence, glucose tolerance (38, 39). Over the last years, a large number of studies investigating VAT Treg cells have uncovered phenotypic and transcriptional features that are peculiar to this unique Treg cell population.

The fraction of eTreg cells among total CD4^+^ T cells in the VAT is age-dependent and reaches a fraction of ~50% in 20-30 week-old male mice, substantially higher than in other lymphoid and non-lymphoid tissues (43). In addition, the TCR repertoire of VAT Treg cells exhibits a greater frequency of shared clonotypes than Treg cells in lymphoid tissues, indicating that the increase in Treg cells in VAT is caused by an antigen-driven clonal expansion of Treg cells (39, 44). This supports the notion that a specific population of Treg cells, most likely PPARγ-low splenic Treg cells, migrates into the VAT and occupies a specific niche where Treg cells can reside over longer periods of time and expand after antigenic stimulation.

By virtue of the proinflammatory environment in the VAT, Treg cells are recruited to the VAT along chemokine gradients, e.g., the chemokine (C-C motif) ligand 2 (CCL2)/CCR2 axis as Treg cells preferentially express CCR2 (38, 39). TCR signaling by oligoclonal Treg cells in the VAT plays a crucial role in their subsequent maturation and acquisition of the canonical VAT Treg cell phenotype. Specifically, TCR signaling in the VAT induces the expression of the transcription factors IRF4 and BATF, which, in turn, elicit the IL-33 receptor, ST2 (45) and enforce expression of the transcription factor PPARγ which is critical for the development and function of VAT Treg cells (32). Meanwhile, stromal cells in the VAT release IL-33, which acts on Treg cell-expressed ST2 to induce VAT Treg cell expansion (38). Mature ST2^+^ VAT Treg cells exhibit a Th2-like phenotype, expressing the prototypical Th2 cell transcription factor GATA3, and releasing IL-13 and IL-5, in addition to IL-10 (46). Together, the interplay of these factors and the signals received by the surrounding microenvironment result in the canonical VAT Treg cell signature comprising, among others, ST2, CCR2, KLRG1, and IL-10 (38, 46). Further, VAT Treg cells adapt to their tissue of residence by upregulating the expression of genes encoding lipid-metabolizing enzymes, such as Pcyt1a (encoding choline-phosphate cytidylyltransferase) and Dgat1 (encoding diacylglycerol O-acyltransferase 1) (32). Notably, ST2^+^ Treg cells do not constitute the entire VAT Treg cell compartment; instead, 20% of the VAT Treg cell population comprises of an interferon-gamma (IFNγ)-dependent C-X-C chemokine receptor type 3 (CXCR3)^+^ Treg cell subset (31, 47, 48). The exact molecular determinants and the population dynamics of such CXCR3^+^ VAT Treg cells remain to be elucidated.

These data, together with additional experimental evidence, supported the idea that the IL-33/ST2 axis is responsible for the recruitment and expansion of Treg cells in the VAT and the development of the VAT Treg cell phenotype (45) and that VAT Treg cells act upon their surroundings *via* secretion of IL-10 (46). Two recent studies have now provided fresh perspectives concerning VAT Treg cell biology, namely the necessity for Treg cell-intrinsic ST2 signaling for VAT Treg cell development and/or maintenance, and the impact of IL-10 released by VAT Treg cells on organismal physiology. On the one hand, deletion of ST2 selectively in Treg cells did not result in decreased numbers of VAT Treg cells or altered expression of KLRG1 and GATA3 (46, 49). Instead, Treg cell-specific ST2 deficiency reduced the capacity of VAT Treg cell to secrete IL-5 and IL-13 (49). Furthermore, ST2-deficiency in Treg cells did not abrogate their expansion in response to IL-33 administration, questioning the hypothesis that IL-33/ST2 is acting in a Treg-cell intrinsic fashion. However, the authors compared mice lacking both *Il1rl1* alleles to *Il1rl1* ^fl/+^ heterozygous mice and not wild-type animals, where IL-33-driven Treg cell expansion might be greater than in *Il1rl1* single-deficient mice (49). On the other hand, Beppu et al. reported that Treg cell-derived IL-10, whose expression is driven by the transcription factor Blimp-1 (38), promotes high-fat diet-induced obesity (50). This was explained by the finding that IL-10 suppresses the differentiation of adipocyte precursors into beige adipocytes, impeding thermogenesis and promoting obesity (50). This suggests that the beneficial role that VAT Treg cells play in guarding against insulin resistance is IL-10-independent. Taken together, these studies highlight that the role and function of VAT Treg cells is still not fully understood and warrants further investigations to unravel the intricate dependencies between the local interplay of cells in the given tissue microenvironment.

However, the vast majority of the aforementioned studies were carried out in male mice. A recent study has now shown that VAT Treg cells exhibit a substantial sexual dimorphism (38). Treg cells were more abundant in VAT of male mice and expressed larger amounts of ST2, KLRG1, CCR2, IL-10, as well as Blimp-1 and PPARγ. The distinct differentiation states of Treg cells in VAT of male and female mice were shaped by sex hormones in a Treg cell-extrinsic fashion (38). Specifically, instead of acting directly on Treg cells, testosterone stimulated the production of pro-inflammatory mediators, including CCL2, and IL-33 by VAT stromal cells in male mice (38). Conversely, estrogen limited the release of such mediators in VAT of female mice. CCR2-expressing Treg cells were recruited to the CCL2-rich male VAT, where they expanded *in situ* by virtue of locally-produced IL-33 acting on Treg-expressed ST2 (38). Taken together, the pronounced inflammatory state of the male VAT facilitates Treg cell residence in VAT to mitigate inflammation and insulin resistance.

In addition to the aforementioned transcriptional regulators and surface receptors, recent studies have illuminated additional signaling pathways that control VAT Treg cell abundance and phenotype. Germline deletion of ICOS was associated with an increased number of VAT Treg cells, which exhibited a more pronounced effector phenotype (48) including higher expression of CTLA-4 and KLRG1. Such accumulation of Treg cells was mirrored by a reduced frequency of Th1 cells and a reciprocal increase of Th2 cells. Remarkably, lack of ICOS signaling resulted in an increased IL-10 production by effector CD4^+^ T cells in the VAT (48). In addition, male mice deficient in ICOS signaling and exposed to HFD exhibited a greater increase in ST2 and KLRG1 expressing eTreg cells in the VAT compared to WT mice, along with an accumulation of Th2-polarized effector CD4^+^ T cells and ameliorated insulin sensitivity (48). Amplification of eTreg cells in the VAT in the absence of ICOS was in disagreement with observations made for Treg cells in lymphoid and other non-lymphoid tissues in the same mice, where Treg cells were reduced in numbers and showed a naive-like phenotype. Hence, one can conclude from this study that ICOS signaling serves to limit eTreg expansion specifically in VAT.

Functionally, recent work could identify VAT-specific immunosuppressive programs. Hydroxyprostaglandin dehydrogenase (HPGD) is an enzyme that mediates the metabolism of prostaglandin E2 (PGE_2_) into 15-keto PGE_2_, which mediates an immunosuppressive effect on Tconv cells (51). Expression of HPGD is higher in Treg cells compared to Tconv cells and is greater in VAT Treg cells compared to other lymphoid and non-lymphoid tissue Treg cells. Notably, HPGD expression is at least partially dependent on FoxP3 and PPARγ. Hpgd-deficient Treg cells retain the transcriptional and phenotypic hallmarks of unperturbed Treg cells. However, Treg-specific Hpgd deficiency in the context of aging or diet-induced obesity resulted in a VAT-specific expansion of functionally impaired Treg cells (51). Consequently, mice harboring Hpgd-deficient Treg cells exhibited an increased infiltration of natural killer (NK) cells and inflammatory CD11c^+^ myeloid cells in the VAT, which culminated in VAT inflammation and an impaired metabolic profile corresponding to reduced insulin sensitivity. These data support that VAT Treg cells not only are transcriptionally distinct but also can use tissue- and context-specific means to exert their function within a given non-lymphoid tissue.

A recent single-cell RNA-seq (scRNA-seq) study has further extended our understanding of VAT Treg cell heterogeneity. Employing a multimodal analysis of adipose tissue Treg cells characterizing their transcriptome, chromatin accessibility, and TCR repertoire, the authors could describe that VAT Treg cells can be broadly classified into two main subsets based on the expression of ST2 and the surface nucleotidase CD73 (44). Notably, the CD73-expressing Treg cells were distinct from the Th1-like CXCR3^+^ Treg cells described above. Rather, the CD73-expressing Treg cell cluster was marked by a naïve phenotype, a reduced expression of effector Treg cell molecules, and a less diverse TCR repertoire compared to the ST2-expressing Treg cell cluster. CD73-expressing Treg cells represent a less differentiated state and trajectory analysis and functional data support that the CD73-expressing Treg cells convert into ST2-expressing Treg cells, a transition that is dependent on insulin signaling. Indeed, insulin signaling promoted the expression of PPARγ, which, in turn, downregulated CD73 and upregulated ST2 expression (44). One potential reason as to why a CXCR3-expressing subset was not identified in this study is the coarse clustering employed by the authors, given that CXCR3^+^ Treg cells only represent a minority in the VAT, and thus would potentially require further subclustering for its detection.

In summary, Treg cells in the VAT represent a vital tool in controlling local and systemic inflammation and promoting insulin sensitivity. They are recruited to the inflammatory environment of the VAT and are then shaped by the tissue microenvironment. VAT Treg cells possess peculiar features compared to other tissue Treg cells with respect to the role of ICOS signaling in their maintenance and sexual dimorphism. In the future, the ongoing characterization of this unique Treg cell population may allow for their precise targeting to prevent and treat metabolic disorders.

## Functions of tissue Treg cells for muscle homeostasis and tissue repair

In 2013 Burzyn et al. for the first time described a small population of skeletal muscle-specific Treg cells. These cells possess unique transcriptional features with genes upregulated that are important for Treg cell-mediated suppressive functions, such as *Ctla4, Klrg1, Il10, Gzmb, Tim-3, Ccr2, Il1rl1*, and *Areg*, as well as genes related to mitotic cell cycle pathways (52–54). Based on high expressions of Helios and Neuropilin-1 (Nrp-1), a thymic origin of this muscle-specific Treg cell population was suggested (53). In response to a cardiotoxin-induced skeletal muscle injury, this Treg cell fraction in mice increased by clonal expansion in the inflamed tissue (53, 55). DiSpirito et al. furthermore confirmed that the Treg cell response after muscle injury is a dynamic process which is marked by specific shifts in the Treg cell transcriptome (54), highlighting the importance and need of longitudinal studies throughout the course of regeneration to better capture these dynamic changes and learn about their importance for Treg cell function during muscle repair.

Muscle injury in general starts with the destruction of muscle fibers, cell death, and infiltration of pro-inflammatory immune cells followed by the activation, proliferation, and differentiation of myogenic stem cells, called satellite cells, promoting tissue repair and regeneration (56). Thereby a shift from pro-inflammatory to anti-inflammatory responses occurs. Interestingly, Treg cells accumulation is most prominent during this shift and is induced in a TCR-dependent manner (53, 57, 58). Similar to the recruitment of Treg cells into VAT, IL-33-ST2 signaling has been proposed as a key pathway necessary for the migration of Treg cells into skeletal muscle. First evidence for this came from the observation of high expression of ST2 on muscle Treg cells and impaired muscle regeneration upon Treg cell-specific deletion of ST2 (55). In such a scenario, the alarmin IL-33 is released by fibro/adipogenic progenitor cells and local mesenchymal stromal cells which are associated with neural structures proposing a crosstalk between the immune and nervous system (55, 59). Furthermore, old mice were shown to secrete lower amounts of IL-33 upon muscle injury, which resulted in a diminished Treg cell fraction within skeletal muscle, as well as impaired muscle repair. This age-dependent distortion of tissue homeostasis could be restored by IL-33 supplementation, further supporting the protective function of Treg cells within skeletal muscle (55). Generally, Treg cell contribution to muscle healing includes macrophage polarization into a pro-regenerative phenotype and direct stimulation of satellite cell proliferation *via* amphiregulin (53, 55, 57, 60). The importance of Treg cells for muscle tissue homeostasis has been further highlighted by several additional studies which showed that genetic deletion or pharmacological abrogation of Treg cells in mice resulted in delayed muscle healing, increased fibrosis, and prolonged inflammation (53, 55, 57, 60)

One additional function of Treg cells in skeletal muscle has been unraveled through studies in the mdx mouse model. This is a murine model of the human disease Duchenne muscular dystrophy characterized as a hallmark by chronic muscle inflammation (61). Comparable to the acute muscle injury, an increase in Treg cells was detected in necrotic lesions of mdx mice. The Treg cells expressed higher levels of KLRG1, GITR, programmed cell death protein 1 (PD-1), and IL-10, reflecting an activated phenotype (53, 55, 57, 60), supporting the notion that, also in chronic muscle damage and inflammation, tissue-specific Treg cells are required to contain tissue inflammation.

Recently, the importance of PD-1 for muscle-Treg cell expansion and function could be established using a skeletal muscle contusion mouse model (62). Another factor shaping the Treg cell population within the skeletal muscle is adenosine triphosphate (ATP), which earlier had been shown to be inhibitory to Treg cell stability and suppressive function (63). During the course of muscle injury, necrotic muscle fibers and immune cells release ATP activating the purinergic P2X receptors on Treg cells leading to their inhibition. The blockage of this pathway in mdx mice resulted in enriched Treg cells within the skeletal muscle and attenuated injury progression (64). As observed during acute muscle injury, pharmacological Treg cell expansion in mdx mice reduced muscle injury and severity of inflammation, while Treg cell depletion exacerbated the damage (53, 55, 57, 60). Summarizing these findings, skeletal muscle harbors a unique Treg cell population which is important for tissue repair and regeneration with further potential aspects e.g., during obesity or other challenges of muscle tissue not yet sufficiently explored.

Besides skeletal muscle, Treg cells have also been described in the cardiac muscle. Such heart tissue Treg cells adopt a specific program helping to maintain tissue homeostasis through dampening chronic inflammation. This has been best studied in heart failure, which is characterized by structural abnormalities and cardiac dysfunction leading to reduced peripheral organ perfusion (65). Myocardial infarction, ischemia/reperfusion injury, as well as myocardial fibrosis may directly lead to heart failure and findings from these ethiopatholigically connected diseases will be summarized here.

Myocardial infarction is marked by low oxygen delivery to the myocardial tissue resulting in cardiomyocyte death. During the tissue repair process cells from the innate immune system, especially neutrophils and monocytes/macrophages, infiltrate the damaged tissue initializing local inflammation. On the other side, Treg cells enrich in the infarcted myocardium, partly mediated *via* CCR5, promoting tissue regeneration (66–69). Treg cells thereby diminish the infiltration of pro-inflammatory cells, mediate macrophage polarization into an anti-inflammatory tissue-repairing phenotype, and directly promote cardiomyocyte proliferation (66–68, 70–72). A recent study by Xia et al. performed RNA-seq of Treg cells seven days post myocardial infarction, revealing a Treg cell subtype being present in the heart that is consistent with a tissue Treg cell phenotype also found in skeletal muscle and skin, characterized by genes such as *Ctla4, Areg, Klrg1*, and *Il1rl1* (68). These cells further upregulated genes which have been mainly described in the context of collagen biosynthesis, wound healing, and extracellular matrix organization, confirming acquisition of a tissue-regenerative function of the tissue Treg cells. Moreover, Treg cells present in the infarcted myocardium have been shown to be actively-recruited and thymus-derived, Helios^hi^Nrp-1^hi^ Treg cells that further expand within the tissue in an IL-33-ST2-dependent manner (68).

Myocardial ischemia/reperfusion injury follows myocardial infarction and may lead to additional damage. In mouse models a rapid Treg cell infiltration of the heart post reperfusion was described (68, 73, 74). While not further characterized on a transcriptional level, it is conceivable to assume that these eTreg cells will have a highly similar tissue-instructed phenotype. Functionally, adoptive transfer of *in vitro*-activated Treg cells attenuated the myocardial ischemia/reperfusion injury in a CD39-dependent manner. Such Treg cells promoted cardiomyocyte survival and inhibited neutrophil infiltration, highlighting one exemplary pathway how tissue homeostasis can be achieved by tissue Treg cells (73).

Myocardial fibrosis occurs as a consequence to cardiomyocyte injury further decreasing cardiac function. Adoptive transfer of Treg cells has been shown to ameliorate the extent of fibrosis in mice (75–78). Conversely, in neonatal mice, where the heart can transiently regenerate after injury, Treg cell depletion following heart cryoinfarction resulted in reduced cardiomyocyte proliferation and enhanced fibrosis (78). Transcriptomic analysis of Treg cells during the regenerative phase after cryoinfarction depicted an upregulation of chemotaxis and repair-related genes such as *Ccl24* and *Areg* (78) further supporting a regenerative Treg cell phenotype being present in the heart. Taken together, these data clearly highlight the importance of a muscle-specific program in Treg cells being recruited into both skeletal and cardiac muscle after injury where these eTreg cells subsequently exhibit a tissue reparative function.

Beyond their role in cardiac muscle repair after myocardial infarction, tissue Treg cells have also been implicated in the context of atherosclerosis, which is one of the main risk factors for myocardial infarction and stroke and is characterized by chronic sterile inflammatory remodeling of the arterial wall (79, 80). This subsequently results in plaque formation inside of arteries that consists of lipids and immune cells such as macrophages, dendritic cells (DCs), or T cells (81) but low frequencies of Treg cells (82–85). The first evidence for atheroprotective functions of Treg cells was reported in mouse studies with either pharmacological depletion of Treg cells using anti-CD25 antibodies (86, 87) or temporal FoxP3-specific diphtheria toxin-targeted (FoxP3^DTR^) (88) depletion of Treg cells in mouse models for atherosclerosis, namely in apolipoprotein E–deficient (*ApoE^–/–^*) or low-density lipoprotein receptor–deficient (*Ldlr^–/–^*) mice. In these mice Treg cell deficiency was associated with elevated lesion development (86–88). Conversely, *ApoE^–/–^* mice supplemented with Treg cells *via* adoptive transfer showed attenuated lesion formation and immune cell infiltration (89, 90). Elucidating the exact suppressive mechanism exerted by Treg cells in atherosclerosis, Lin et al. studied macrophage foam-cell formation, a hallmark of atherosclerosis, in presence or absence of Treg cells (91). It could be shown that Treg cells *ex vivo* decrease lipid accumulation and promote an anti-inflammatory phenotype of co-cultured macrophages. Requirements for this protective effect were both direct cell-cell contact as well as soluble factors, like the anti-inflammatory cytokines IL-10 and TGF-β. The importance of IL-10 and TGF-β for attenuating atherosclerosis was subsequently confirmed by additional studies (92–96). However, Treg cells are not the exclusive source for these cytokines, other cell types such as DCs or type 1 regulatory T (Tr1) cells need to be taken into consideration as contributors to this atheroprotective mechanism. In the context of regression of atherosclerotic lesions, Sharma et al. further characterized Treg cell subtypes involved in disease development and resolution using scRNA-seq of CD45^+^ aortic cells. Based on the expression of Nrp-1, Treg cells from progressing plaques were identified as thymus-derived, while Treg cells in regressing plaques were mainly induced in the periphery, had a higher activation status, and an altered metabolism (87). It remains open, what factors cause this change, is the migration into the tissue altered or is this change dependent on tissue-antigens being presented and what functional impact has this altered Treg cell phenotype for the local tissue environment. A recent study in *ApoE^–/–^* mice by Shao et al. shed some further light on the Treg cells involved in atherosclerosis demonstrating that IL-35 promotes a subset of CCR5^+^ Treg cells, which are characterized by elevated expression of immunosuppressive genes such as *TIGIT, Pdcd1, Ctla4, Adora2a, Lag3, Havcr2*, and *Il10.* This tissue Treg cell population is important for the prevention of the formation of atheroscerotic lesions (97). Taken together, these studies could unravel the importance of Treg cells within the context of atherosclerosis and demonstrate that the eTreg cells mainly exhibit atheroprotective properties. Current data suggests however, that this activity is carried out by different Treg cell subsets over the course of the disease which warrants further investigations to better understand the impact of these subtypes of Treg cells. Important first steps would be to characterize these cells on a transcriptional and functional level to align findings with current knowledge on eTreg cell properties.

## Role of Treg cells for CNS development and homeostasis

While the contribution of Treg cells to tissue homeostasis in adipose tissue and muscle have been well documented over the last decade, focus has shifted to the role of Treg cells for tissue homeostasis in other not as-well studied organ systems, such as the central nervous system (CNS). Several studies over the last few years have reshaped our perception of the immune cell composition in the CNS. Single-cell profiling revealed that the brain immune cell landscape is heterogeneous and comprises several subsets of myeloid and lymphoid cells (98) under steady-state and pathophysiological conditions beyond autoimmunity and infection. Recent work has described that Tconv cells but also Treg cells can take up residency in the brain parenchyma at steady state, where they contribute to the maturation of microglia and hence to brain homeostasis (99). In addition, CD4^+^ T cells have been described in the meninges, particularly in the dura, and implicated in the modulation of cognition and behavior (100). In young adult mice, Treg cells represent ~10% of the CD4^+^ T cell population in both the brain parenchyma and dura (99, 101). The majority of brain Treg cells were characterized by an eTreg cell phenotype, expressing elevated amounts of CD73, CD39, and lymphocyte-activation gene 3 (LAG3), and displayed similar suppressive capacity *in vitro* compared to their splenic counterparts (102). Interestingly, astrocytes were found to maintain FoxP3 expression in Treg cells and promoted Treg cell survival in an IL-2*/*Signal transducer and activator of transcription 5 (STAT5)-dependent fashion (102). Much like the expansion of Treg cells in peripheral tissues of old mice (103), meningeal Treg cells likewise increase in frequencies and numbers in the dura of old mice (101). Systemic depletion of Treg cells, including in the dura and deep cervical lymph nodes, mitigated the cognitive impairment exhibited by old mice, hence indirectly implicating Treg cells in age-related cognitive deficits (101).

A number of studies have characterized brain Treg cells in the context of ischemic stroke (104–107). Treg cells infiltrate the brain following the induction of stroke and increase in numbers particularly at later time points (day >7 post-stroke induction) compared to the acute and subacute phases (3-7 days) post-stroke induction (105, 107). Treg cells in the ischemic brain, but not in peripheral tissues, were highly proliferative 7-14 days after ischemia (105); they localized close to and within the infarcted area, where they contact resident and infiltrating MHC-II^+^ myeloid cells (104, 105). Brain Treg cells are recruited to the ischemic brain *via* chemokines such as CCL1 and CCL20 which act on CCR6 and CCR8 expressed by Treg cells (106). Subsequently, Treg cells are expanded in a ST2-dependent fashion, with IL-33 released from injured glia acting on ST2 expressed by Treg cells (106). In addition to typical markers of tissue Treg cells (106, 107), brain Treg cells in the ischemic brain exhibited context-dependent gene expression, in the shape of serotonin receptor Htr7 (106), In line with this finding, Treg cell expansion was dependent on serotonin in the ischemic brain (106). Treg cell depletion resulted in an enhanced disruption of white-matter integrity, reduced capacity for remyelination, and impaired neurological recovery (106, 107). Of note, Treg cell depletion did not aggravate motor functions of mice in the acute phases of stroke (105, 107), suggesting a restorative potential of Treg cells at later time points rather than an anti-inflammatory effect during the early phases of injury. Mechanistically, Treg cells mediate their reparative functions on remyelination and oligodendrogenesis by modulating microglial gene expression profiles rather than inhibiting pro-inflammatory effector T cell response (107). Specifically, a Treg cell-microglia crosstalk mediated by Treg cell-bound osteopontin and microglia-expressed integrin subunit beta 1 (Itgb1) was shown to be instrumental for promoting a reparative program in microglia (107). Another mode of action of Treg cells in ischemic stroke has recently been described as they can mitigate the stroke-associated astrogliosis in an amphiregulin-dependent fashion and suppress the induction of a neurotoxic gene expression program in astrocytes (106). Evidence also exists for a role of Treg cells in the context of intracerebral hemorrhage (ICH). Treg cells increased in the spleen and brain of mice following ICH induction, albeit to a greater extent in the brain, compared to sham-operated mice (108). Treg cell depletion two days before the induction of ICH was associated with aggravated neuroinflammation, hematoma volume, neuronal death, and motor impairment, suggesting that Treg cells also mediate tissue integrity and protection in this setting (108).

Brain Treg cells have also been investigated in another context of tissue injury, namely traumatic brain injury (TBI) (109). TBI elicited a greater expression of IL-33 by astrocytes and oligodendrocytes. This was accompanied by an increased infiltration of ST2^+^ Treg cells into the brain, particularly on day 7 post-TBI. Mice with constitutive *Il1rl1* deletion exhibited reduced numbers of circulating and brain-infiltrating Treg cells, a reduction that was correlated with worsened histopathological findings and neurological impairment (109). Conversely, IL-33 treatment was associated with increased ST2^+^ Treg numbers in the brain and ameliorated neuropathological and behavioral scores (109). Importantly, IL-33 administration to Treg cell-depleted mice did not lead to neurological improvements observed in IL-33-treated Treg-sufficient mice (109). Hence, IL-33 release from injured neural cells promotes a recovery program mediated by ST2^+^ Treg cells.

Furthermore, Treg cells mitigate neuropathic pain preferentially in female mice (110). Intrathecal administration of colony-stimulating factor 1 (CSF-1), and subsequent microglia activation, precipitates neuropathic pain specifically in male mice. In female mice, intrathecal CSF-1 delivery was associated with an expansion of Treg cells (and NK cells) in the meninges lining the spinal cord. Systemic depletion of Treg cells in female mice followed by intrathecal CSF-1 injection resulted in microglia adopting a transcriptional landscape similar to the state observed in male mice, together with the induction of pain in such female mice, highlighting a sex-specific protective function of CNS Treg cells in female animals (110). Another example of sexual dimorphism of brain Treg cells was observed in the setting of hypoxia-induced encephalopathy in neonates (111). Brain Treg cells were more numerous in the brains of female mice 24 hours post induction of hypoxia, expressing the chemokine receptors CCR4 and CXCR4. Depletion of Treg cells in female mice exacerbated neuropathology as well as motor and behavioral impairments (111). Strikingly, Treg depletion in male neonatal mice mitigated neuronal injury and motor and cognitive deficits. Mechanistically, Treg cells from female mice exhibited greater suppressive capacity of effector T cells, and their depletion resulted in enhanced microglial and endothelial cell activation in female but not male mice (111).

Beyond their role for acute injury and repair, Treg cells have also been implicated in the context of neurodegeneration. In 6 week-old amyloid precursor protein/presenilin 1 (APP/PS1) mice as model for cerebral amyloidosis, transient depletion of Treg cells impaired microglial clustering around amyloid β (Aβ) plaques and aggravated cognitive decline, whereas IL-2 administration induced Treg cell expansion and ameliorated cognition (112). In contrast, Treg cell depletion in 5 month-old 5x Familial Alzheimer’s Disease (FAD) mice, as a second model of cerebral amyloidosis, enhanced myeloid cell trafficking to the brain and improved cognitive function (113). While it would be tempting to speculate on a protective role for Treg cells in neurodegeneration, these opposing results which may be attributed to the different age - and hence different extent of disease progression - at which Treg cells were depleted showcase that there still exists a knowledge gap on the role of Treg cells in the context of neurodegeneration that has to be addressed over the upcoming years.

As discussed in previous sections, tissue Treg cells and their molecular machinery adopt tissue- and context-specificities. Similarly, it is conceivable that two common tissue Treg cell markers, ST2 and ICOS, modulate brain Treg cells as so far already demonstrated in the context of stroke but also CNS autoimmunity and infection. Besides their identification as crucial for repair after brain injury, ST2^+^ Treg cells in the brain have been recently characterized in the context of CNS autoimmunity, namely mice developing experimental autoimmune encephalomyelitis (EAE), a model of autoimmune neuroinflammation. Conditional deletion of ST2 in Treg cells resulted in increased frequency and absolute number of Treg cells in the CNS of EAE mice (49). Despite this change in frequency, deletion of ST2 in Treg cells resulted in more severe EAE manifestations; moreover, gamma-delta T cells were more numerous and secreted more IL-17A but less IFNγ after depletion of ST2 in Treg cells. Therefore, although the frequency and absolute number of Treg cells were higher in mice lacking ST2 expression in Treg cells, the authors suggested that the lack of signaling *via* ST2 seemed to impede the modulation of pathogenic, IL-17-producing gamma-delta T cells by Treg cells further highlighting the importance of ST2 for function and proper positioning of Treg cells in the CNS (49). ICOS was also implicated as a regulator of the maintenance of brain Treg cells. Using a model of brain Treg cell expansion, namely chronic infection with the parasite Toxoplasma gondii (114), ICOS deficiency abrogated the increased proportion of Treg cells otherwise observed in chronically infected wild type mice (47). This is in line with data demonstrating a reduced frequency of Treg cells in the spleen and most non-lymphoid tissues in ICOS-deficient mice at steady state, with the notable exception of VAT, as discussed above. A functional consequence of the impaired Treg cell expansion in the brain of chronically infected mice could not be inferred due to the use of non-conditional ICOS deficient mice, hence affecting - in addition - Tconv cell responses. Still, these data support that both ST2 and ICOS contribute vitally in shaping the CNS Treg cell phenotype.

Collectively, these studies highlight a tissue reparative role for CNS Treg cells following tissue injury-induced innate inflammation, but also a detrimental role in aging and later stages of cerebral amyloidosis. In line with findings from other tissues, the data also suggest that core aspects of the program of CNS tissue Treg cells molecularly and functionally overlap with tissue Treg cells from other tissues while still adopting context-specific features as e.g., expression of Htr7 showcases.

## Hepatic Treg cell and their contribution to liver homeostasis

The liver is another organ in which Treg cells have been implicated in health and disease. Under homeostatic conditions, Treg cells represent 4-8% of the hepatic CD4^+^ T cell compartment in young adult mice (115, 116). We will cover here how hepatic Treg cells in neonates are shaped by the gut-liver axis and how Treg cells orchestrate tissue remodeling upon liver injury in adulthood. For a more thorough overview of Treg cell implication in liver health and disease, the reader is referred to the following reviews (117–119).

Treg cell recruitment into the liver can be observed already quite early after birth. Specifically, Treg cells represent 15% of the CD4^+^ T cell compartment in the mouse liver during the first 2 weeks of age, a frequency that drops to <5% in young adult mice. This increase of liver Treg cells in neonates was mediated by the combination of TGF-β signaling and intestinal colonization by microbiota and subsequent transient MyD88 signaling secondary to colonization (120). Depletion of Treg cells during postnatal week 2 resulted in increased systemic levels of pro-inflammatory cytokines, but surprisingly tissue injury was most pronounced in the liver compared with other non-lymphoid tissues (121). Indeed, such abrogation of Treg cell expansion in neonates and the ensuing hepatic tissue injury, precipitated a dysregulated glucose metabolism in the liver, leading to impaired mouse growth (121). Consistent with other non-lymphoid tissue Treg cells, liver Treg cells comprised more effector and less naïve Treg cells compared to their splenic counterparts. In addition, liver Treg cells in neonatal mice displayed higher proliferation rates in comparison to splenic Treg cells, however hepatic Treg cells also showed higher apoptosis rate (121), indicating that the population of hepatic Treg cells is highly active but only short-lived. Transcriptional characterization of hepatic Treg cells revealed that canonical features of other tissue Treg cells, namely the expression of ST2, KLRG1, PPARγ, as well as lipid metabolism enzymes, were also found in liver but not in spleen Treg cells (121). Furthermore, similar to e.g., VAT, one-third of the liver Treg cell pool was characterized by a Th1-like transcriptome and phenotype, suggesting that also in the liver several different tissue Treg cell populations can exist and might be associated with distinct functional properties.

Following neonatal expansion of hepatic Treg cells and their subsequent contraction, the diminished pool of adult Treg cells also has important roles for liver homeostasis. Hepatic Treg cells establish and maintain tolerance to non-pathogenic foreign antigens, such as nutrients, delivered from the gut *via* the portal circulation (122). Indeed, dietary antigen-specific hepatic Treg cells produce elevated amounts of IL-10 upon encountering their cognate antigens and prevent mounting of a cytotoxic CD8^+^ T cell response against such antigens (122). This is in stark contrast to splenic Treg cells which are unable to upregulate IL-10 under the same conditions, highlighting again the specific features Treg cells gain when differentiating towards an effector phenotype in a tissue.

Similar to their orchestrating role in sterile inflammation in the VAT, hepatic Treg cells have also been implicated in the context of non-alcoholic steatohepatitis (NASH). High-fat and high-carbohydrate diets, which can induce NASH, were associated with an increased number and proliferation rate of Treg cells in the liver (123). This was mirrored by increased fractions of IFNγ and TNFα expressing T cells in the total hepatic CD4^+^ T cell compartment (123). Notably, adoptive transfer of Treg cells to mice post-feeding them a high-fat, high-sugar diet exacerbated liver steatosis and liver cell injury (123, 124). This is in contrast to an earlier study documenting a detrimental impact on liver injury upon Treg cell depletion (125). Important differences between these studies include the duration during which mice received a high-fat diet and the genetic background, demonstrating that additional work is needed to understand how tissue Treg cells contribute to tissue repair in NASH.

In addition, hepatic Treg cells mitigate the course of chronic inflammation-induced liver fibrosis. Induction of chronic inflammation in the liver by administration of carbon tetrachloride (CCl4) resulted in an increased proportion of Treg cells in the liver but not in lymphoid tissues (126). In line with the findings from other tissues, hepatic Treg cells in CCl4-treated mice exhibited an elevated expression of ST2, consistent with an enhanced release of IL-33 upon cell injury. In addition, Treg cell depletion combined with CCl4 treatment favored the establishment of a profibrotic environment in the liver, characterized by an elevated Th2/Th1 cell ratio and an increased infiltration of Ly6C^+^CCR2^+^ myeloid cells, culminating in exacerbated liver fibrosis (126). Conversely, Treg cell depletion without CCl4 only resulted in acute liver injury without the development of fibrosis. Importantly, and unlike e.g., muscle or brain tissue Treg cells (106), Treg cell-mediated hepatic tissue protection was amphiregulin-independent, highlighting the context dependence of Treg cell-mediated tissue repair. Finally, a recent study has implicated the IL–33/ST2 axis in the resolution of heightened hepatic inflammation in the context of LPS-induced sepsis (127), namely that IL-33 released from injured liver cells recruits ST2^+^ Treg cells, which then facilitate the resolution of inflammation.

Taken together, hepatic Treg cells follow a dynamic trajectory of seeding and residing in the liver during young age. Liver Treg cells share common features with other subsets, both transcriptionally and functionally, including recruitment and expansion *via* the IL-33/ST2 axis as well as tissue repair function, while at the same time exhibiting context-specific functionalities, as evidenced by the induction of tolerance to dietary antigens.

## Contribution of tissue Treg cells to kidney homeostasis

Under steady state conditions, the murine kidney houses different subsets of T cells, with the majority of them exhibiting a phenotype of antigen-experienced, tissue-resident T cells (128). The fraction of Treg cells among the renal CD4^+^ T cell population in young adult mice was estimated to be 4-15% (128–131), although studies varied in the sex of mice used and the employment of transcardiac perfusion to eliminate circulating leukocytes. In fact, studies in rats point to a greater number of Treg cells in female rat kidneys compared to male rats (132). Of note, kidney-related sex discrepancies were previously reported, with females being more likely to survive following an acute kidney injury (133), and less likely to develop hypertension, as discussed below.

A large number of studies have investigated the protective role of Treg cells in the context of acute kidney injury (AKI). Two prominent models for AKI, which primarily precipitates an innate immune cell response, are cisplatin administration and ischemia-reperfusion injury (IRI). Cisplatin directly induces renal epithelial cell death, with subsequent release of danger-associated molecular patterns (DAMPs) that engage toll-like receptors (TLRs) on renal parenchymal cells. Such engagement stimulates the production of pro-inflammatory cytokines and the recruitment of a plethora of immune cells to the kidneys (134). On the other hand, ischemic renal injury followed by reperfusion of the kidney induces a largely similar sequence of events as observed with cisplatin nephrotoxicity (135). In line with their well-known role in tissue repair, renal Treg cells modulate renal tissue fibrosis in the context of IRI. do Valle Duraes et al. compared the renal CD4^+^ T cell compartment in two IRI models of divergent outcomes, where the ischemic kidney either restores its normal function and architecture (“regeneration” model) or develops tissue fibrosis (133). Renal Treg cells expanded in both IRI models compared to control mice, with a more pronounced expansion, particularly at later time points post-injury. Treg cells from both AKI contexts as well as unchallenged mice exhibited an overlap in the expression of the core tissue Treg cell signature (e.g., *Areg, Itgae* (encoding CD103)*, Ctla4, Tnfrsf4*), whereas the expression of *Il1rl1* and *Klrg1* were largely confined to Treg cells from injured kidney. Importantly, Treg cells from fibrotic kidneys were marked by the expression of genes associated with inflammation and apoptosis (e.g., *Junb, Id2, Gata3*), whereas Treg cells in regenerating kidneys upregulated the expression of genes associated with angiogenesis (e.g., *Nrp-1, Vegfa*). Thus, the underlying tissue injury and ensuing inflammatory milieu dictated variable Treg cell expression profiles and functions (133). Finally, expansion of renal Treg cells by means of IL-2 and IL-33 administration prior to AKI induction mitigated the development of renal tissue damage and fibrosis and the extent of body weight loss, although it was not assessed whether this translated into preserved renal function (133). It is important to note that male and female mice were used in the fibrosis and regeneration models, respectively, with the different sexes potentially contributing to the variable extent of inflammation observed in the two models (133). In an additional study, amplification of Treg cells, systemically and in the kidneys by virtue of IL-2 and IL-33 co-administration before IRI induction mitigated the severity of tubular injury and myeloid cell infiltration into the kidneys and rescued renal functions (136). A confounding factor in this context is the concurrent expansion of innate lymphoid cell (ILC)2 by the same cytokines; hence the protective effect of IL-2/IL-33 against IRI might not be exclusively mediated by Treg cells (136). In the context of cisplatin-induced AKI, Treg cell depletion prior to cisplatin administration aggravated tubular injury and deteriorated renal clearance (137). Conversely, Treg cell transfer mitigated the course of cisplatin-induced renal injury by inhibiting the cisplatin-driven innate immune activation (137). Notably, Treg cell-mediated protection against cisplatin nephrotoxicity was IL-10-independent (138). Further, Treg cell recruitment to and maintenance in renal tissue following cisplatin administration was dependent on TLR9 signaling, which modulates the expression of adhesion molecules CD44 and CD11a by Treg cells, facilitating their recruitment to the kidneys (139). This was nicely demonstrated by the fact that TLR9-deficient Treg cells were not impaired in their capacity to suppress Tconv cells *ex vivo*, produce IL-10 or TGF-β, or express CTLA-4 and CD73. Taken together these data support the notion that Treg cells in the kidney after AKI mainly have a protective function and contribute to functional tissue repair and maintenance of kidney function after tissue damage.

Renal Treg cells were also characterized in a mouse model of glomerulonephritis, where antibodies targeting glomerular basement membrane proteins are transferred into mice. Glomerulonephritis induced in this fashion was associated with a long-term amplification of kidney Treg cells post injury. Treg cell depletion 21 days post-disease induction resulted in further impairment of renal function with a concomitant increase in Th1 cells (140). Kidney-infiltrating Treg cells displayed a molecular profile resembling that of other non-lymphoid tissue Treg cells, namely the expression of ST2, KLRG1, PPARγ, and IL-10 (140). In particular, GATA3-expressing Treg cells represented the majority of Treg cells found in the kidney post induction of glomerulonephritis and expressed higher amounts of Treg cell effector molecules compared to GATA3-negative Treg cells. Administration of the PPARγ agonist pioglitazone increased the fraction of GATA3-expressing cells among kidney Treg cells without altering the total number of Treg cells. Co-transfer of disease-inducing Tconv cells into T cell-deficient mice with either wild-type Treg cells or Treg cells lacking GATA3 emphasized a critical role for GATA3 in controlling kidney injury through affecting the differentiation of tissue Treg cells in the kidney (140).

The kidney controls systemic blood pressure, and one mechanism of such control is by regulating electrolyte absorption and clearance. Early studies using adoptive Treg cell transfer experiments have implicated Treg cells in the renal control of blood pressure (141, 142). Focusing on endogenous renal Treg cells, female rats exhibited greater frequencies of renal Treg cells compared to male rats, both in unchallenged as well as hypertensive rats. Induction of hypertension in rats was associated with a reduced frequency of renal Treg cells compared to sex-matched controls, particularly in female rats. Consequently, depletion of Treg cells resulted in an elevation of blood pressure only in female rats (132). This increased number of Treg cells in the kidneys of female spontaneously hypertensive rats (SHR) was not a reflection of a more frequent systemic Treg cell population in female rats, as assessed in the spleens of male and female SHR (143). Instead, such differences in renal Treg cells across sexes emanated from a greater recruitment of Treg cells to the kidneys of female mice, since surgical removal of the spleen led to a reduction in renal Treg cells and increases in blood pressure in male and female SHR (143). In line with the observed increase of Treg cells in the kidney in hypertension, mice receiving a high-salt diet exhibited an increased number of Treg cells in the kidneys and small intestine, which was accompanied by an elevated number of Tconv cells (131). From *in vitro* studies showing that high-salt levels induced the expression of retinoic acid receptor-related orphan receptor γt (RORγt) in Treg cells with a concomitant reduction in the expression of FoxP3 and Helios and the observation that the Treg cells in this model *in vivo* mainly expressed RORγt one might conclude that in this model the function of Treg cells might also be altered. Indeed, Helios-negative Treg cells, which represent a minority among thymic Treg cells, are more likely to adopt a Th17-like phenotype supporting altered function of these cells in high-salt diet-induced hypertension in the kidney. However, these transcriptional alterations neither drove the expression of IL17a nor mitigated the functional fitness of Treg cells (131). Without additional studies further addressing this question in more detail, the functional role of these Treg cells remains open.

Taken together, these studies provide evidence on a largely beneficial role for renal Treg cells in varying contexts of renal tissue injury, whereby Treg cells suppress inflammation and promote tissue repair. They also implicate Treg cells in blood pressure control, a vital process of which the kidney is a central regulator.

## Tissue Treg cells in reproductive organs

To date little is known about Treg cells in the reproductive tract. Most studies have focused on pregnancy as Treg cells play a crucial role for pregnancy success, providing immunological tolerance to the fetus (144). To support this, Treg cells expand in the endometrium and decidua induced by elevated human chorionic gonadotropin (hCG) levels after fertilization (145), peak during the second trimester, and finally decrease towards birth (146). Salvany-Celades et al. further investigated the phenotype of Treg cells in the decidua during pregnancy, suggesting the existence of three distinct Treg cell subtypes: CD25^hi^FoxP3^+^HELIOS^+^ Treg cells, PD-1^hi^IL-10^+^ Treg cells and TIGIT^+^FoxP3^dim^ Treg cells, whereby mainly the PD-1^hi^IL-10^+^ Treg cell subset actively contributed to effector T cell suppression (147). The great importance of Treg cells in preventing maternal inflammatory responses during pregnancy can be further hypothesized based on reduced decidual Treg cell proportions in spontaneous abortion patients (148, 149) and miscarriage cases (150, 151). In line with these findings, infertile women show reduced endometrial FoxP3 mRNA expression (152). Besides playing an important role in the endometrium during homeostasis and pregnancy, Treg cells have also been described in endometriosis. Endometriosis is a gynecological disease associated with the implantation and development of ectopic endometrial tissues partly mediated by alterations in the normal immune response (153). Several human studies addressed the presence of Treg cells in eutopic and ectopic endometrium showing an overall tendency towards an expansion of Treg cells in endometriosis lesions (154–156). Depending on the grade of severity, a further elevation was observed in women with more severe endometriosis (157, 158). To further elucidate the importance of Treg cells for disease progression, Tanaka et al. temporarily depleted Treg cells in a murine model of endometriosis using FoxP3^DTR^ mice which resulted in elevated number and weight of endometrial lesions, suggesting that the tissue Treg cells have a protective role (159). In contrast to these findings, Xiao et al. showed in a recent study that Treg cell depletion by anti-CD25 antibody treatment reduced the weight of lesions in mice (160). Despite the discrepancies in outcome, these findings showcase that tissue Treg cells have importance for tissue homeostasis in the endometrium and warrant further investigations to better understand their contributions to tissue maintenance and regeneration.

Besides their role in the endometrium, also in the endocervix elevated levels of FoxP3^+^CTLA-4^+^ Treg cells have been described and support a protective role of Tregs cells also for the endocervix, as their numbers are negatively correlated to pro-inflammatory cytokines levels in the tissue and contribute to reduced genital tract inflammation (161).

In the mammary gland the tissue is reorganized during the different reproductive stages (162). This is reflected in alterations of the immune cell landscape which resembles other mucosal tissues. In the nulliparous mammary gland, Treg cells are almost absent and only slightly increase during pregnancy and lactation. Only after lactation and weaning, an extension of RORγt^+^ FoxP3^+^ Treg cells has been reported (162). This could be caused by the epithelial cell death occurring during this period which will lead to increased self-antigens being released which these tissue Treg cells might counterbalance and further support tissue repair.

Similar to the protective function of Treg cells in other female reproductive organs, a role for tissue Treg cells for ovary gland homeostasis/physiology has been suggested (163). While their functional and transcriptional phenotype has not been addressed so far in greater detail, current data support a protective function as in a murine model for premature ovarian insufficiency adoptive transfer of Treg cells could alleviate ovarian cell apoptosis in a protein kinase B (Akt) signaling-dependent manner (164).

Several reports have also established the presence of Treg cells in the testis under homeostatic conditions (165–167). Similarly to other non-lymphoid tissues, Treg cells in the testis express CD25 and FoxP3 (168), feature a memory phenotype, and produce TGF-β (169). Functionally, these eTreg cells are characterized by their recognition of spermatic antigens preventing proliferation of auto-reactive Tconv cells *in vitro* (169), suggesting that these eTreg cells recognize and suppress reactivity against antigens from the seminiferous tubules *in vivo*. This finding is supported by recent data that could show that in the testis non-sequestered germ cell antigens can egress from seminiferous tubules and after entering the interstitium contribute to the induction of Treg cells (170). This observation clearly challenges the prevailing view that all germ cell neo-antigens are sequestered from the immune system and will not be presented under homeostatic conditions. Treg cells induced against these antigens are exerting suppression against auto-reactive Tconv cells maintaining a tolerogenic environment (169, 170).

Taken together these data further support that tissue Treg cells are present within the reproductive tract and exert suppression even against germ-line antigens, however their transcriptional make-up and unique peculiarities are so far not defined and need to be assessed in more detail. Similarly, how Treg cells impact e.g., the different phases of the menstrual cycle or the menopause are not investigated so far.

## Overview of tissue Treg cell function at surface barriers

The gastrointestinal tract, skin, and lung are the main surface barriers protecting the hosts against infection. Treg cells exhibit different functions within these tissues with the overall goal to maintain homeostasis. We will highlight here several recent findings and refer the reader to more exhaustive reviews for a thorough discussion of Treg cell function and more general aspects of tissue Treg cell biology in gut, skin, and lung (2, 171–177).

In the gut two distinct Treg cell phenotypes can be observed: GATA3-expressing Treg cells (178) and RORγt-expressing Treg cells (179). RORγt^+^ Treg cell differentiation is dependent on c-Maf (180, 181) and occurs in the periphery in response to microbiota and food antigens (171, 179, 182–184). RORγt^+^ Treg cells control local inflammatory responses as well as the microbiota balance (180). In contrast, GATA3^+^ Treg cells are thymus-derived and support tissue repair in an IL-33-dependent pathway (185), similar to other ST2-expressing tissue Treg cell populations. Recently, another thymic-derived Treg cell population in the gastrointestinal tract has been described which is characterized by the expression of the transcription factor Zbtb20 (186). These cells are highly active (marked by elevated expressions of CD44, KLRG1, TIGIT, GITR, and ICOS), secrete IL-10, and expand in response to inflammation. In general, gut Treg cells are responsible for the maintenance of tolerance against food-antigens and microbial products, as well as the mucosal barrier while being able to contribute to tissue repair through the expression of amphiregulin.

Also in the skin, Treg cells have been described and contribute to tissue homeostasis. Recently, Miragaia et al. followed up on the question of tissue adaptation of Treg cells in barrier tissues such as colon and skin using single-cell transcriptomics (187). Analysis of the scRNA-seq data supported the idea of a lymph node-to-tissue developmental axis based on the pseudotime trajectory analysis of the Treg cell compartment (187). In addition, a core tissue Treg cell signature could be established from Treg cells isolated from non-lymphoid tissues marked by genes of the TNFRSF-NF-κB signaling pathway as well as genes associated with an effector phenotype such as *Klrg1, Cd44*, or *Il10*. Comparison with transcriptome data from gut Treg cells revealed that these cells resemble the GATA3^+^ Treg cells found in the gastrointestinal tract and are the main population of skin Treg cells (187). Developmentally, it could be shown that Treg cells accumulate in the skin during the neonatal period of life exhibiting an elevated expression of the transcription factor GATA3 (188, 189). Skin-resident Treg cells promote immune tolerance to commensal bacteria (188), contribute to tissue repair mediated *via* the epidermal growth factor receptor (EGFR) (190), and facilitate hair regeneration by promoting anagen induction *via* Jagged1 (191) supporting again a role of tissue Treg cells for repair and regeneration under non-inflammatory conditions.

Similar to the other surface barrier organs, the lung harbors a thymus-derived Treg cell population characterized by Nrp-1, Helios, GATA3, ST2, and TIGIT expression (192). IL-33 *via* interaction with ST2 is an important mediator for these cells. It is not only required for their suppressive phenotype, but as recently shown by Kanno et al., IL-33 is also involved in the regulation of Treg cell metabolism. IL-33 thereby induces the expression of Acyl-CoA synthetase (Acsbg1), an enzyme important for fatty acid oxidation in the mitochondria, which further promotes the resolution of IL-33-induced airway inflammation (193). While this finding clearly suggests that factors governing tissue Treg cell biology can impact the metabolism of Treg cells, the metabolic requirements of tissue-resident Treg cells are largely unknown. Moreover, recent data stress the importance to study these aspects *in vivo* using genetic model systems. This is e.g. highlighted by studies where pharmacological inhibition of fatty acid transport into mitochondria *via* the enzyme carnitine palmitoyltransferase 1 (CPT1) by etomoxir was shown to decrease Treg cell development *in vitro* (194), while genetic FoxP3-specific *Cpt1a* ablation resulted in similar Treg cell numbers with unchanged FoxP3 expression levels compared to wild type controls (195). Given the discrepancies between the experimental systems used, it will be important in the future to also study the metabolism of tissue Treg cells in greater detail in its tissue context to evaluate its importance for tissue Treg cell differentiation and function.

One leading pathology causing lung tissue damage is chronic obstructive pulmonary disease (COPD) resulting in chronic inflammation and tissue remodeling leading to severe airflow limitations. Numbers of Treg cells in lung tissues or bronchoalveolar lavage samples from COPD patients are decreased in comparison to healthy controls (196–198). This observation could be confirmed in mice which were exposed to cigarette smoke further revealing an additional imbalance in the Th17/Treg cell ratio (199–201). Unfortunately, so far data is missing on the exact role and phenotype of tissue Treg cells in COPD.

More research has been performed on the involvement of tissue Treg cells in acute lung injury (ALI). ALI is usually defined by an uncontrolled inflammatory response followed by a resolution and regeneration phase which is to a certain degree facilitated by Treg cells. Intratracheal lipopolysaccharide (LPS) induces alveolar epithelial damage and is therefore used as a common mouse model for ALI. Using this model, D’Alessio et al. described an enrichment of Treg cells in the lung during the resolution phase. Abrogation of Treg cells delayed the resolution of the lung injury while adoptive transfer of Treg cells into *Rag-1^-/-^* animals facilitated the repair accompanied by decreased pro-inflammatory cytokines, increased neutrophil clearance, and elevated TGF-β levels (202). Treg cells contribute *via* different mechanisms to the resolution phase. For example, Treg cells directly promote epithelial proliferation in an CD103-dependent manner (203). Moreover, Treg cells produce growth factors promoting wound healing: keratinocyte growth factor and amphiregulin, both factors are known to further stimulate epithelial proliferation (204). Amphiregulin thereby serves a central role for the Treg cell-mediated tissue repair. Arpaia et al. identified amphiregulin-producing Treg cells by elevated expressions of CD44, GITR, CTLA-4, KLRG-1, PD-1, and CD103 highlighting their effector phenotype (205). Interestingly, amphiregulin expression in Treg cells was not required for their suppressive function and its production was independent of TCR-engagement. Instead, amphiregulin expression relied on the IL-1 family cytokines IL-18 and IL-33 (205). The alarmin IL-33 has been additionally shown as an important factor for the resolution of inflammation by the induction of IL-10 and IL-13 and reduction of pro-inflammatory responses in ALI (206). CD73-dependent generation of adenosine by Treg cells was discovered to be another functional pathway for Treg cell-mediated resolution of lung injury (207). Transcriptional analysis of Treg cells from the lung during resolution of ALI revealed a distinct transcriptomic profile compared to splenic Treg cells marked by upregulation of genes such as *Areg*, *Il1rl1*, *Il18r1, Itgae*, *Ctla4*, *Icos*, and *Il10* confirming the previously described mechanisms for resolution of ALI while at the same time recapitulating several common characteristics of tissue Treg cells (208). In some patients ALI can lead to pulmonary fibrosis defined by extracellular matrix protein deposition within the lung tissue. On one hand, Lo Re et al. described a pro-fibrotic function of Treg cells directly stimulating fibroblast proliferation and collagen deposition in a platelet-derived growth factor–B (PDGF-B)-TGF-β-dependent manner using a silica (SiO_2_)-induced mouse model for fibrosis (209). On the other hand, Garibaldi et al. showed that Treg cells reduce the C-X-C Motif Chemokine Ligand 12 (CXCL12)/CXCR4-mediated recruitment of fibrocytes to the lung thereby ameliorating fibrosis after LPS injury in *Rag-1^-/-^* mice (210). These two studies again demonstrate the difficulties in characterizing tissue Treg cell function as the discrepancies of these two studies may result from differences in the experimental system and time points of analysis. Therefore, to better understand the exact contribution of tissue Treg cells to pulmonary fibrosis further studies are warranted.

Taken together, also in barrier organs, tissue Treg cells contribute to tissue homeostasis and repair, with recent single-cell data further helping to understand the developmental pathways towards acquisition of the transcriptional tissue Treg cell program.

## Concluding remarks and future perspectives

In the past years, there has been a steadily growing interest in understanding the contribution of Treg cells to non-lymphoid tissue homeostasis. Recent work has revealed the existence of tissue-specific Treg cells with an effector phenotype which play profound roles in tissue maintenance, regeneration, and repair. While the regenerative function is shared across different tissues, unique tissue-specific Treg cell properties have been identified. This can be nicely exemplified by the distinct transcriptional signatures characterizing the different tissue Treg cells. While they share common effector Treg cell gene modules, Treg cells in each tissue also express tissue-specific programs instructed by the tissue microenvironment and available growth factor milieu.

Although we have begun to understand many aspects of tissue Treg cell biology, certain aspects remain to be described in greater detail to build a comprehensive model of the plethora of tissue Treg cells. This includes further work on the origin of Treg cells within different tissues and the developmental steps leading to tissue Treg cells differentiation, including the specific TCR repertoire required within each tissue. Furthermore, their transcriptional and metabolic adaptations to the new tissue microenvironment, which is governed by e.g., availability of oxygen and nutrients or communication with resident cells will be important to describe and understand. Despite the difficulties, technological advances such as single-cell technologies provide new opportunities in answering these remaining questions.

Recently, sex discrepancies have been described for tissues such as VAT (38), CNS (110, 111), or the kidneys (133, 143) revealing a knowledge gap that needs to be addressed in future investigations. This is particularly evident from the findings in VAT where sex hormones actually influence Treg cell differentiation and phenotype. If this finding can also be confirmed for other tissues, we will need to revisit some of the current concepts of the role of tissue Treg cells for tissue homeostasis. Similarly, differences for Treg cell accumulation within peripheral tissues throughout life have been detected (103). Characterizing Treg cell phenotypes as well as functional properties in both sexes at different ages in non-lymphoid tissues would be therefore required to further current paradigms of tissue Treg cell biology. Generally, such variations in the experimental layout may explain existing contradictory observations and a deeper transparency in the experimental outline of the conducted studies would be important for better data comparison.

Finally, this review outlined the importance of tissue-specific Treg cells in non-lymphoid tissue physiology with a deeper insight into different pathophysiological conditions. The translation of the wealth of data from animal studies into humans will be an important next step in the future. This could help to identify properties which are required for Treg cells in a given tissue and could as such be harnessed to equip chimeric antigen receptor (CAR)-Treg cells with these properties to use them in a therapeutic setting (211).

## Author contributions

All authors wrote and revised the manuscript. All authors contributed to the article and approved the submitted version.

## Funding

MB, DM, and TE were funded by the Deutsche Forschungsgemeinschaft (DFG, German Reseach Foundation) - project number 272482170 - GRK2168.

## Conflict of interest

The authors declare that the research was conducted in the absence of any commercial or financial relationships that could be construed as a potential conflict of interest.

## Publisher’s note

All claims expressed in this article are solely those of the authors and do not necessarily represent those of their affiliated organizations, or those of the publisher, the editors and the reviewers. Any product that may be evaluated in this article, or claim that may be made by its manufacturer, is not guaranteed or endorsed by the publisher.

## References

[1] Josefowicz SZ Lu LF Rudensky AY. Regulatory T cells: mechanisms of differentiation and function. Annu Rev Immunol (2012) 30:531–64. doi: 10.1146/annurev.immunol.25.022106.141623 PMC6066374 22224781

[2] Panduro M Benoist C Mathis D. Tissue tregs. Annu Rev Immunol (2016) 34:609–33. doi: 10.1146/annurev-immunol-032712-095948 PMC4942112 27168246

[3] Nakagawa H Wang L Cantor H Kim HJ. New insights into the biology of CD8 regulatory t cells. Adv Immunol (2018) 140:1–20. doi: 10.1016/bs.ai.2018.09.001 30366517

[4] Cunnusamy K Baughman EJ Franco J Ortega SB Sinha S Chaudhary P. Disease exacerbation of multiple sclerosis is characterized by loss of terminally differentiated autoregulatory CD8+ T cells. Clin Immunol (2014) 152:115–26. doi: 10.1016/j.clim.2014.03.005 PMC4024444 24657764

[5] Baughman EJ Mendoza JP Ortega SB Ayers CL Greenberg BM Frohman EM. Neuroantigen-specific CD8+ regulatory T-cell function is deficient during acute exacerbation of multiple sclerosis. J Autoimmun (2011) 36:115–24. doi: 10.1016/j.jaut.2010.12.003 PMC3046327 21257291

[6] Kim HJ Verbinnen B Tang X Lu L Cantor H. Inhibition of follicular T-helper cells by CD8(+) regulatory T cells is essential for self tolerance. Nature (2010) 467:328–32. doi: 10.1038/nature09370 PMC3395240 20844537

[7] Owen DL Mahmud SA Sjaastad LE Williams JB Spanier JA Simeonov DR. Thymic regulatory T cells arise *via* two distinct developmental programs. Nat Immunol (2019) 20:195–205. doi: 10.1038/s41590-018-0289-6 30643267 PMC6650268

[8] Hori S Haury M Coutinho A Demengeot J. Specificity requirements for selection and effector functions of CD25+4+ regulatory T cells in anti-myelin basic protein T cell receptor transgenic mice. Proc Natl Acad Sci USA (2002) 99:8213–8. doi: 10.1073/pnas.122224799 PMC123047 12034883

[9] Fontenot JD Rasmussen JP Williams LM Dooley JL Farr AG Rudensky AY. Regulatory T cell lineage specification by the forkhead transcription factor foxp3. Immunity (2005) 22:329–41. doi: 10.1016/j.immuni.2005.01.016 15780990

[10] Jordan MS Boesteanu A Reed AJ Petrone AL Holenbeck AE Lerman MA. Thymic selection of CD4+CD25+ regulatory T cells induced by an agonist self-peptide. Nat Immunol (2001) 2:301–6. doi: 10.1038/86302 11276200

[11] Hsieh CS Liang Y Tyznik AJ Self SG Liggitt D Rudensky AY. Recognition of the peripheral self by naturally arising CD25+ CD4+ T cell receptors. Immunity (2004) 21:267–77. doi: 10.1016/j.immuni.2004.07.009 15308106

[12] Salomon B Lenschow DJ Rhee L Ashourian N Singh B Sharpe A. B7/CD28 costimulation is essential for the homeostasis of the CD4+CD25+ immunoregulatory T cells that control autoimmune diabetes. Immunity (2000) 12:431–40. doi: 10.1016/s1074-7613(00)80195-8 10795741

[13] Tai X Cowan M Feigenbaum L Singer A. CD28 costimulation of developing thymocytes induces Foxp3 expression and regulatory T cell differentiation independently of interleukin 2. Nat Immunol (2005) 6:152–62. doi: 10.1038/ni1160 15640801

[14] Chen W Jin W Hardegen N Lei KJ Li L Marinos N. Conversion of peripheral CD4+CD25- naive T cells to CD4+CD25+ regulatory T cells by TGF-beta induction of transcription factor FoxP3. J Exp Med (2003) 198:1875–86. doi: jem.20201142/jem.20030152 10.1084/jem.20030152 PMC2194145 14676299

[15] Kretschmer K Apostolou I Hawiger D Khazaie K Nussenzweig MC von Boehmer H. Inducing and expanding regulatory T cell populations by foreign antigen. Nat Immunol (2005) 6:1219–27. doi: 10.1038/ni1265 16244650

[16] Gottschalk RA Corse E Allison JP. And affinity determine peripheral induction of Foxp3 *in vivo* . J Exp Med (2010) 207:1701–11. doi: 10.1084/jem.20091999 PMC2916126 20660617

[17] Bettelli E Carrier Y Gao W Korn T Strom TB Oukka M. Reciprocal developmental pathways for the generation of pathogenic effector TH17 and regulatory T cells. Nature (2006) 441:235–8. doi: 10.1038/nature04753 16648838

[18] Tone Y Furuuchi K Kojima Y Tykocinski ML Greene MI Tone M. Smad3 and NFAT cooperate to induce Foxp3 expression through its enhancer. Nat Immunol (2008) 9:194–202. doi: 10.1038/ni1549 18157133

[19] Xu L Kitani A Stuelten C McGrady G Fuss I Strober W. Positive and negative transcriptional regulation of the Foxp3 gene is mediated by access and binding of the Smad3 protein to enhancer I. Immunity (2010) 33:313–25. doi: 10.1016/j.immuni.2010.09.001 PMC2972198 20870174

[20] D’Cruz LM Klein L. Development and function of agonist-induced CD25+Foxp3+ regulatory T cells in the absence of interleukin 2 signaling. Nat Immunol (2005) 6:1152–9. doi: 10.1038/ni1264 16227983

[21] Fontenot JD Rasmussen JP Gavin MA Rudensky AY. A function for interleukin 2 in Foxp3-expressing regulatory T cells. Nat Immunol (2005) 6:1142–51. doi: 10.1038/ni1263 16227984

[22] Antony PA Paulos CM Ahmadzadeh M Akpinarli A Palmer DC Sato N. Interleukin-2-dependent mechanisms of tolerance and immunity *in vivo* . J Immunol (2006) 176:5255–66. doi: 10.4049/jimmunol.176.9.5255 PMC1473163 16621991

[23] Laurence A Tato CM Davidson TS Kanno Y Chen Z Yao Z. Interleukin-2 signaling *via* STAT5 constrains T helper 17 cell generation. Immunity (2007) 26:371–81. doi: 10.1016/j.immuni.2007.02.009 17363300

[24] Davidson TS DiPaolo RJ Andersson J Shevach EM. Cutting edge: IL-2 is essential for TGF-beta-mediated induction of Foxp3+ T regulatory cells. J Immunol (2007) 178:4022–6. doi: 10.4049/jimmunol.178.7.4022 17371955

[25] Mucida D Park Y Kim G Turovskaya O Scott I Kronenberg M. Reciprocal TH17 and regulatory T cell differentiation mediated by retinoic acid. Science (2007) 317:256–60. doi: 10.1126/science.1145697 17569825

[26] Mangan PR Harrington LE O’Quinn DB Helms WS Bullard DC Elson CO. Transforming growth factor-beta induces development of the T(H)17 lineage. Nature (2006) 441:231–4. doi: 10.1038/nature04754 16648837

[27] Cretney E Kallies A Nutt SL. Differentiation and function of Foxp3(+) effector regulatory T cells. Trends Immunol (2013) 34:74–80. doi: 10.1016/j.it.2012.11.002 23219401

[28] Cretney E Xin A Shi W Minnich M Masson F Miasari M. The transcription factors blimp-1 and IRF4 jointly control the differentiation and function of effector regulatory T cells. Nat Immunol (2011) 12:304–11. doi: 10.1038/ni.2006 21378976

[29] Kawabe T Jankovic D Kawabe S Huang Y Lee PH Yamane H. Memory-phenotype CD4(+) T cells spontaneously generated under steady-state conditions exert innate TH1-like effector function. Sci Immunol (2017) 2. doi: 10.1126/sciimmunol.aam9304 PMC5568832 28783663

[30] Yang BH Wang K Wan S Liang Y Yuan X Dong Y. TCF1 and LEF1 control treg competitive survival and tfr development to prevent autoimmune diseases. Cell Rep (2019) 27:3629–45.e6. doi: 10.1016/j.celrep.2019.05.061 31216480 PMC6701704

[31] Li C DiSpirito JR Zemmour D Spallanzani RG Kuswanto W Benoist C. TCR transgenic mice reveal stepwise, multi-site acquisition of the distinctive fat-treg phenotype. Cell (2018) 174:285–99.e12. doi: 10.1016/j.cell.2018.05.004 29887374 PMC6046274

[32] Cipolletta D Feuerer M Li A Kamei N Lee J Shoelson SE. PPAR-gamma is a major driver of the accumulation and phenotype of adipose tissue treg cells. Nature (2012) 486:549–53. doi: 10.1038/nature11132 PMC3387339 22722857

[33] Li C Munoz-Rojas AR Wang G Mann AO Benoist C Mathis D. PPARgamma marks splenic precursors of multiple nonlymphoid-tissue treg compartments. Proc Natl Acad Sci USA (2021) 118 (13):e2025197118. doi: 10.1073/pnas.2025197118 33753509 PMC8020779

[34] Delacher M Imbusch CD Hotz-Wagenblatt A Mallm JP Bauer K Simon M. Precursors for nonlymphoid-tissue treg cells reside in secondary lymphoid organs and are programmed by the transcription factor BATF. Immunity (2020) 52:295–312.e11. doi: 10.1016/j.immuni.2019.12.002 31924477 PMC7026712

[35] Delacher M Simon M Sanderink L Hotz-Wagenblatt A Wuttke M Schambeck K. Single-cell chromatin accessibility landscape identifies tissue repair program in human regulatory T cells. Immunity (2021) 54:702–20.e17. doi: 10.1016/j.immuni.2021.03.007 33789089 PMC8050210

[36] Sullivan JM Hollbacher B Campbell DJ. Cutting edge: Dynamic expression of id3 defines the stepwise differentiation of tissue-resident regulatory t cells. J Immunol (2019) 202:31–6. doi: 10.4049/jimmunol.1800917 PMC6311998 30518568

[37] Khan S Chan YT Revelo XS Winer DA. The immune landscape of visceral adipose tissue during obesity and aging. Front Endocrinol (Lausanne) (2020) 11:267. doi: 10.3389/fendo.2020.00267 32499756 PMC7243349

[38] Vasanthakumar A Chisanga D Blume J Gloury R Britt K Henstridge DC. Sex-specific adipose tissue imprinting of regulatory T cells. Nature (2020) 579:581–5. doi: 10.1038/s41586-020-2040-3 PMC7241647 32103173

[39] Feuerer M Herrero L Cipolletta D Naaz A Wong J Nayer A. Lean, but not obese, fat is enriched for a unique population of regulatory T cells that affect metabolic parameters. Nat Med (2009) 15:930–9. doi: 10.1038/nm.2002 PMC3115752 19633656

[40] Fox CS Massaro JM Hoffmann U Pou KM Maurovich-Horvat P Liu CY. Abdominal visceral and subcutaneous adipose tissue compartments: association with metabolic risk factors in the framingham heart study. Circulation (2007) 116:39–48. doi: 10.1161/CIRCULATIONAHA.106.675355 17576866

[41] Strissel KJ DeFuria J Shaul ME Bennett G Greenberg AS Obin MS. T-Cell recruitment and Th1 polarization in adipose tissue during diet-induced obesity in C57BL/6 mice. Obes (Silver Spring) (2010) 18:1918–25. doi: 10.1038/oby.2010.1 PMC2894258 20111012

[42] Suganami T Nishida J Ogawa Y. A paracrine loop between adipocytes and macrophages aggravates inflammatory changes: role of free fatty acids and tumor necrosis factor alpha. Arterioscler Thromb Vasc Biol (2005) 25:2062–8. doi: 10.1161/01.ATV.0000183883.72263.13 16123319

[43] Cipolletta D Cohen P Spiegelman BM Benoist C Mathis D. Appearance and disappearance of the mRNA signature characteristic of treg cells in visceral adipose tissue: age, diet, and PPARgamma effects. Proc Natl Acad Sci USA (2015) 112:482–7. doi: 10.1073/pnas.1423486112 PMC4299242 25550516

[44] Li Y Lu Y Lin SH Li N Han Y Huang Q. Insulin signaling establishes a developmental trajectory of adipose regulatory T cells. Nat Immunol (2021) 22:1175–85. doi: 10.1038/s41590-021-01010-3 34429546

[45] Vasanthakumar A Moro K Xin A Liao Y Gloury R Kawamoto S. The transcriptional regulators IRF4, BATF and IL-33 orchestrate development and maintenance of adipose tissue-resident regulatory T cells. Nat Immunol (2015) 16:276–85. doi: 10.1038/ni.3085 25599561

[46] Siede J Frohlich A Datsi A Hegazy AN Varga DV Holecska V. IL-33 receptor-expressing regulatory t cells are highly activated, Th2 biased and suppress CD4 T cell proliferation through IL-10 and TGFbeta release. PLoS One (2016) 11:e0161507. doi: 10.1371/journal.pone.0161507 27548066 PMC4993514

[47] O’Brien CA Harris TH. ICOS-deficient and ICOS YF mutant mice fail to control toxoplasma gondii infection of the brain. PLoS One (2020) 15:e0228251. doi: 10.1371/journal.pone.0228251 31978191 PMC6980566

[48] Mittelsteadt KL Hayes ET Campbell DJ. ICOS signaling limits regulatory T cell accumulation and function in visceral adipose tissue. J Exp Med (2021) 218(6):e20201142. doi: 10.1084/jem.20201142 33881452 PMC8065270

[49] Hemmers S Schizas M Rudensky AY. T Reg cell-intrinsic requirements for ST2 signaling in health and neuroinflammation. J Exp Med (2021) 218(2):e20201234. doi: 10.1084/jem.20201234 33095261 PMC7590508

[50] Beppu LY Mooli RGR Qu X Marrero GJ Finley CA Fooks AN. Tregs facilitate obesity and insulin resistance *via* a blimp-1/IL-10 axis. JCI Insight (2021) 6(3):e140644. doi: 10.1172/jci.insight.140644 PMC7934851 33351782

[51] Schmidleithner L Thabet Y Schonfeld E Kohne M Sommer D Abdullah Z. Enzymatic activity of HPGD in treg cells suppresses tconv cells to maintain adipose tissue homeostasis and prevent metabolic dysfunction. Immunity (2019) 50:1232–48.e14. doi: 10.1016/j.immuni.2019.03.014 31027998

[52] Ronin E Pouchy C Khosravi M Hilaire M Gregoire S Casrouge A. Tissue-restricted control of established central nervous system autoimmunity by TNF receptor 2-expressing treg cells. Proc Natl Acad Sci USA (2021) 118(13):e2014043118. doi: 10.1073/pnas.2014043118 33766913 PMC8020675

[53] Burzyn D Kuswanto W Kolodin D Shadrach JL Cerletti M Jang Y. A special population of regulatory T cells potentiates muscle repair. Cell (2013) 155:1282–95. doi: 10.1016/j.cell.2013.10.054 PMC3894749 24315098

[54] DiSpirito JR Zemmour D Ramanan D Cho J Zilionis R Klein AM. Molecular diversification of regulatory T cells in nonlymphoid tissues. Sci Immunol (2018) 3(27):eaat5861. doi: 10.1126/sciimmunol.aat5861 30217811 PMC6219455

[55] Kuswanto W Burzyn D Panduro M Wang KK Jang YC Wagers AJ. Poor repair of skeletal muscle in aging mice reflects a defect in local, interleukin-33-Dependent accumulation of regulatory T cells. Immunity (2016) 44:355–67. doi: 10.1016/j.immuni.2016.01.009 PMC4764071 26872699

[56] Tidball JG. Mechanisms of muscle injury, repair, and regeneration. Compr Physiol (2011) 1:2029–62. doi: 10.1002/cphy.c100092 23733696

[57] Castiglioni A Corna G Rigamonti E Basso V Vezzoli M Monno A. FOXP3+ T cells recruited to sites of sterile skeletal muscle injury regulate the fate of satellite cells and guide effective tissue regeneration. PLoS One (2015) 10:e0128094. doi: 10.1371/journal.pone.0128094 26039259 PMC4454513

[58] Cho J Kuswanto W Benoist C Mathis D. T Cell receptor specificity drives accumulation of a reparative population of regulatory T cells within acutely injured skeletal muscle. Proc Natl Acad Sci U.S.A. (2019) 116(52):26727–26733. doi: 10.1073/pnas.1914848116 PMC6936428 31822623

[59] Wang K Yaghi OK Spallanzani RG Chen X Zemmour D Lai N. Neuronal, stromal, and T-regulatory cell crosstalk in murine skeletal muscle. Proc Natl Acad Sci USA (2020) 117:5402–8. doi: 10.1073/pnas.1922559117 PMC7071852 32102913

[60] Villalta SA Rosenthal W Martinez L Kaur A Sparwasser T Tidball JG. Regulatory T cells suppress muscle inflammation and injury in muscular dystrophy. Sci Transl Med (2014) 6:258ra142. doi: 10.1126/scitranslmed.3009925 PMC4889432 25320234

[61] Nitahara-Kasahara Y Takeda S Okada T. Inflammatory predisposition predicts disease phenotypes in muscular dystrophy. Inflammation Regen (2016) 36:14. doi: 10.1186/s41232-016-0019-0 PMC5725653 29259687

[62] Shou J Shi X Liu X Chen Y Chen P Xiao W. Programmed death-1 promotes contused skeletal muscle regeneration by regulating treg cells and macrophages. Lab Invest (2021) 101:719–32. doi: 10.1038/s41374-021-00542-4 PMC8137453 33674785

[63] Schenk U Frascoli M Proietti M Geffers R Traggiai E Buer J. ATP inhibits the generation and function of regulatory T cells through the activation of purinergic P2X receptors. Sci Signal (2011) 4:ra12. doi: 10.1126/scisignal.2001270 21364186

[64] Gazzerro E Baldassari S Assereto S Fruscione F Pistorio A Panicucci C. Enhancement of muscle t regulatory cells and improvement of muscular dystrophic process in mdx mice by blockade of extracellular ATP/P2X axis. Am J Pathol (2015) 185:3349–60. doi: 10.1016/j.ajpath.2015.08.010 26465071

[65] Schwinger RHG. Pathophysiology of heart failure. Cardiovasc Diagn Ther (2021) 11:263–76. doi: 10.21037/cdt-20-302 PMC7944197 33708498

[66] Weirather J Hofmann UD Beyersdorf N Ramos GC Vogel B Frey A. Foxp3+ CD4+ T cells improve healing after myocardial infarction by modulating monocyte/macrophage differentiation. Circ Res (2014) 115:55–67. doi: 10.1161/CIRCRESAHA.115.303895 24786398

[67] Tang TT Yuan J Zhu ZF Zhang WC Xiao H Xia N. Regulatory T cells ameliorate cardiac remodeling after myocardial infarction. Basic Res Cardiol (2012) 107:232. doi: 10.1007/s00395-011-0232-6 22189560

[68] Xia N Lu Y Gu M Li N Liu M Jiao J. A unique population of regulatory T cells in heart potentiates cardiac protection from myocardial infarction. Circulation (2020) 142:1956–73. doi: 10.1161/CIRCULATIONAHA.120.046789 32985264

[69] Dobaczewski M Xia Y Bujak M Gonzalez-Quesada C Frangogiannis NG. CCR5 signaling suppresses inflammation and reduces adverse remodeling of the infarcted heart, mediating recruitment of regulatory T cells. Am J Pathol (2010) 176:2177–87. doi: 10.2353/ajpath.2010.090759 PMC2861083 20382703

[70] Zeng Z Yu K Chen L Li W Xiao H Huang Z. Interleukin-2/Anti-Interleukin-2 immune complex attenuates cardiac remodeling after myocardial infarction through expansion of regulatory t cells. J Immunol Res (2016) 2016:8493767. doi: 10.1155/2016/8493767 27144181 PMC4837274

[71] Jia D Jiang H Weng X Wu J Bai P Yang W. Interleukin-35 promotes macrophage survival and improves wound healing after myocardial infarction in mice. Circ Res (2019) 124:1323–36. doi: 10.1161/CIRCRESAHA.118.314569 30832557

[72] Zacchigna S Martinelli V Moimas S Colliva A Anzini M Nordio A. Paracrine effect of regulatory T cells promotes cardiomyocyte proliferation during pregnancy and after myocardial infarction. Nat Commun (2018) 9:2432. doi: 10.1038/s41467-018-04908-z 29946151 PMC6018668

[73] Xia N Jiao J Tang TT Lv BJ Lu YZ Wang KJ. Activated regulatory T-cells attenuate myocardial ischaemia/reperfusion injury through a CD39-dependent mechanism. Clin Sci (Lond) (2015) 128:679–93. doi: 10.1042/CS20140672 25558978

[74] Saxena A Dobaczewski M Rai V Haque Z Chen W Li N. Regulatory T cells are recruited in the infarcted mouse myocardium and may modulate fibroblast phenotype and function. Am J Physiol Heart Circ Physiol (2014) 307:H1233–42. doi: 10.1152/ajpheart.00328.2014 PMC4200341 25128167

[75] Cao Y Xu W Xiong S. Adoptive transfer of regulatory T cells protects against coxsackievirus B3-induced cardiac fibrosis. PLoS One (2013) 8:e74955. doi: 10.1371/journal.pone.0074955 24023968 PMC3762771

[76] Kvakan H Kleinewietfeld M Qadri F Park JK Fischer R Schwarz I. Regulatory T cells ameliorate angiotensin II-induced cardiac damage. Circulation (2009) 119:2904–12. doi: 10.1161/CIRCULATIONAHA.108.832782 19470887

[77] Kanellakis P Dinh TN Agrotis A Bobik A. CD4(+)CD25(+)Foxp3(+) regulatory T cells suppress cardiac fibrosis in the hypertensive heart. J Hypertens (2011) 29:1820–8. doi: 10.1097/HJH.0b013e328349c62d 21785365

[78] Li J Yang KY Tam RCY Chan VW Lan HY Hori S. Regulatory T-cells regulate neonatal heart regeneration by potentiating cardiomyocyte proliferation in a paracrine manner. Theranostics (2019) 9:4324–41. doi: 10.7150/thno.32734 PMC6599663 31285764

[79] Spitz C Winkels H Burger C Weber C Lutgens E Hansson GK. Regulatory T cells in atherosclerosis: critical immune regulatory function and therapeutic potential. Cell Mol Life Sci (2016) 73:901–22. doi: 10.1007/s00018-015-2080-2 PMC11108393 26518635

[80] Albany CJ Trevelin SC Giganti G Lombardi G Scotta C. Getting to the heart of the matter: The role of regulatory T-cells (Tregs) in cardiovascular disease (CVD) and atherosclerosis. Front Immunol (2019) 10:2795. doi: 10.3389/fimmu.2019.02795 31849973 PMC6894511

[81] Hansson GK Hermansson A. The immune system in atherosclerosis. Nat Immunol (2011) 12:204–12. doi: 10.1038/ni.2001 21321594

[82] de Boer OJ van der Meer JJ Teeling P van der Loos CM van der Wal AC. Low numbers of FOXP3 positive regulatory T cells are present in all developmental stages of human atherosclerotic lesions. PLoS One (2007) 2:e779. doi: 10.1371/journal.pone.0000779 17712427 PMC1945014

[83] Depuydt MAC Prange KHM Slenders L Ord T Elbersen D Boltjes A. Microanatomy of the human atherosclerotic plaque by single-cell transcriptomics. Circ Res (2020) 127:1437–55. doi: 10.1161/CIRCRESAHA.120.316770 PMC7641189 32981416

[84] Dietel B Cicha I Voskens CJ Verhoeven E Achenbach S Garlichs CD. Decreased numbers of regulatory T cells are associated with human atherosclerotic lesion vulnerability and inversely correlate with infiltrated mature dendritic cells. Atherosclerosis (2013) 230:92–9. doi: 10.1016/j.atherosclerosis.2013.06.014 23958259

[85] Rohm I Atiskova Y Drobnik S Fritzenwanger M Kretzschmar D Pistulli R. Decreased regulatory T cells in vulnerable atherosclerotic lesions: imbalance between pro- and anti-inflammatory cells in atherosclerosis. Mediators Inflamm (2015) 2015:364710. doi: 10.1155/2015/364710 25684861 PMC4312649

[86] Ait-Oufella H Salomon BL Potteaux S Robertson AK Gourdy P Zoll J. Natural regulatory T cells control the development of atherosclerosis in mice. Nat Med (2006) 12:178–80. doi: 10.1038/nm1343 16462800

[87] Sharma M Schlegel MP Afonso MS Brown EJ Rahman K Weinstock A. Regulatory T cells license macrophage pro-resolving functions during atherosclerosis regression. Circ Res (2020) 127:335–53. doi: 10.1161/CIRCRESAHA.119.316461 PMC7367765 32336197

[88] Klingenberg R Gerdes N Badeau RM Gistera A Strodthoff D Ketelhuth DF. Depletion of FOXP3+ regulatory T cells promotes hypercholesterolemia and atherosclerosis. J Clin Invest (2013) 123:1323–34. doi: 10.1172/JCI63891 PMC3582120 23426179

[89] Mor A Planer D Luboshits G Afek A Metzger S Chajek-Shaul T. Role of naturally occurring CD4+ CD25+ regulatory T cells in experimental atherosclerosis. Arterioscler Thromb Vasc Biol (2007) 27:893–900. doi: 10.1161/01.ATV.0000259365.31469.89 17272749

[90] Feng J Zhang Z Kong W Liu B Xu Q Wang X. Regulatory T cells ameliorate hyperhomocysteinaemia-accelerated atherosclerosis in apoE-/- mice. Cardiovasc Res (2009) 84:155–63. doi: 10.1093/cvr/cvp182 19502284

[91] Lin J Li M Wang Z He S Ma X Li D. The role of CD4+CD25+ regulatory T cells in macrophage-derived foam-cell formation. J Lipid Res (2010) 51:1208–17. doi: 10.1194/jlr.D000497 PMC2853448 20007839

[92] Pinderski LJ Fischbein MP Subbanagounder G Fishbein MC Kubo N Cheroutre H. Overexpression of interleukin-10 by activated T lymphocytes inhibits atherosclerosis in LDL receptor-deficient mice by altering lymphocyte and macrophage phenotypes. Circ Res (2002) 90:1064–71. doi: 10.1161/01.res.0000018941.10726.fa 12039795

[93] Caligiuri G Rudling M Ollivier V Jacob MP Michel JB Hansson GK. Interleukin-10 deficiency increases atherosclerosis, thrombosis, and low-density lipoproteins in apolipoprotein e knockout mice. Mol Med (2003) 9:10–7. doi: 10.1007/BF03402102 PMC1430379 12765335

[94] Frutkin AD Otsuka G Stempien-Otero A Sesti C Du L Jaffe M. TGF-[beta]1 limits plaque growth, stabilizes plaque structure, and prevents aortic dilation in apolipoprotein e-null mice. Arterioscler Thromb Vasc Biol (2009) 29:1251–7. doi: 10.1161/ATVBAHA.109.186593 PMC2740721 19325140

[95] Robertson AK Rudling M Zhou X Gorelik L Flavell RA Hansson GK. Disruption of TGF-beta signaling in T cells accelerates atherosclerosis. J Clin Invest (2003) 112:1342–50. doi: 10.1172/JCI18607 PMC228445 14568988

[96] Gojova A Brun V Esposito B Cottrez F Gourdy P Ardouin P. Specific abrogation of transforming growth factor-beta signaling in T cells alters atherosclerotic lesion size and composition in mice. Blood (2003) 102:4052–8. doi: 10.1182/blood-2003-05-1729 12920022

[97] Shao Y Yang WY Saaoud F Ct D Sun Y Xu K. IL-35 promotes CD4+Foxp3+ tregs and inhibits atherosclerosis *via* maintaining CCR5-amplified treg-suppressive mechanisms. JCI Insight (2021) 6(19):e152511. doi: 10.1172/jci.insight.152511 34622804 PMC8525592

[98] Mrdjen D Pavlovic A Hartmann FJ Schreiner B Utz SG Leung BP. High-dimensional single-cell mapping of central nervous system immune cells reveals distinct myeloid subsets in health, aging, and disease. Immunity (2018) 48:380–95.e6. doi: 10.1016/j.immuni.2018.01.011 29426702

[99] Pasciuto E Burton OT Roca CP Lagou V Rajan WD Theys T. Microglia require CD4 T cells to complete the fetal-to-Adult transition. Cell (2020) 182:625–40.e24. doi: 10.1016/j.cell.2020.06.026 32702313 PMC7427333

[100] Kipnis J Gadani S Derecki NC. Pro-cognitive properties of T cells. Nat Rev Immunol (2012) 12:663–9. doi: 10.1038/nri3280 PMC4032225 22903149

[101] Da Mesquita S Herz J Wall M Dykstra T de Lima KA Norris GT. Aging-associated deficit in CCR7 is linked to worsened glymphatic function, cognition, neuroinflammation, and beta-amyloid pathology. Sci Adv (2021) 7(21):eabe4601. doi: 10.1126/sciadv.abe4601 34020948 PMC8139596

[102] Xie L Choudhury GR Winters A Yang SH Jin K. Cerebral regulatory T cells restrain microglia/macrophage-mediated inflammatory responses *via* IL-10. Eur J Immunol (2015) 45:180–91. doi: 10.1002/eji.201444823 PMC4293323 25329858

[103] Nishioka T Shimizu J Iida R Yamazaki S Sakaguchi S. CD4+CD25+Foxp3+ T cells and CD4+CD25-Foxp3+ T cells in aged mice. J Immunol (2006) 176:6586–93. doi: 10.4049/jimmunol.176.11.6586 16709816

[104] Liesz A Suri-Payer E Veltkamp C Doerr H Sommer C Rivest S. Regulatory T cells are key cerebroprotective immunomodulators in acute experimental stroke. Nat Med (2009) 15:192–9. doi: 10.1038/nm.1927 19169263

[105] Stubbe T Ebner F Richter D Engel O Klehmet J Royl G. Regulatory T cells accumulate and proliferate in the ischemic hemisphere for up to 30 days after MCAO. J Cereb Blood Flow Metab (2013) 33:37–47. doi: 10.1038/jcbfm.2012.128 22968321 PMC3597367

[106] Ito M Komai K Mise-Omata S Iizuka-Koga M Noguchi Y Kondo T. Brain regulatory T cells suppress astrogliosis and potentiate neurological recovery. Nature (2019) 565:246–50. doi: 10.1038/s41586-018-0824-5 30602786

[107] Shi L Sun Z Su W Xu F Xie D Zhang Q. Treg cell-derived osteopontin promotes microglia-mediated white matter repair after ischemic stroke. Immunity (2021) 54:1527–42.e8. doi: 10.1016/j.immuni.2021.04.022 34015256 PMC8282725

[108] Zhou K Zhong Q Wang YC Xiong XY Meng ZY Zhao T. Regulatory T cells ameliorate intracerebral hemorrhage-induced inflammatory injury by modulating microglia/macrophage polarization through the IL-10/GSK3beta/PTEN axis. J Cereb Blood Flow Metab (2017) 37:967–79. doi: 10.1177/0271678X16648712 PMC5363473 27174997

[109] Xie D Miao W Xu F Yuan C Li S Wang C. IL-33/ST2 axis protects against traumatic brain injury through enhancing the function of regulatory T cells. Front Immunol (2022) 13:860772. doi: 10.3389/fimmu.2022.860772 35432343 PMC9006950

[110] Kuhn JA Vainchtein ID Braz J Hamel K Bernstein M Craik V. Regulatory T-cells inhibit microglia-induced pain hypersensitivity in female mice. Elife (2021) 10:e69056. doi: 10.7554/eLife.69056 34652270 PMC8639143

[111] Beckmann L Obst S Labusek N Abberger H Koster C Klein-Hitpass L. Regulatory T cells contribute to sexual dimorphism in neonatal hypoxic-ischemic brain injury. Stroke (2022) 53:381–90. doi: 10.1161/STROKEAHA.121.037537 PMC8785522 34983246

[112] Dansokho C Ait Ahmed D Aid S Toly-Ndour C Chaigneau T Calle V. Regulatory T cells delay disease progression in Alzheimer-like pathology. Brain (2016) 139:1237–51. doi: 10.1093/brain/awv408 26912648

[113] Baruch K Rosenzweig N Kertser A Deczkowska A Sharif AM Spinrad A. Breaking immune tolerance by targeting Foxp3(+) regulatory T cells mitigates alzheimer’s disease pathology. Nat Commun (2015) 6:7967. doi: 10.1038/ncomms8967 26284939 PMC4557123

[114] O’Brien CA Overall C Konradt C O’Hara Hall AC Hayes NW Wagage S. CD11c-expressing cells affect regulatory T cell behavior in the meninges during central nervous system infection. J Immunol (2017) 198:4054–61. doi: 10.4049/jimmunol.1601581 PMC5451665 28389591

[115] Bai Y Guan F Zhu F Jiang C Xu X Zheng F. IL-33/ST2 axis deficiency exacerbates hepatic pathology by regulating treg and th17 cells in murine schistosomiasis japonica. J Inflammation Res (2021) 14:5981–98. doi: 10.2147/JIR.S336404 PMC8604654 34815688

[116] Zhu H Liu Z An J Zhang M Qiu Y Zou MH. Activation of AMPKalpha1 is essential for regulatory T cell function and autoimmune liver disease prevention. Cell Mol Immunol (2021) 18:2609–17. doi: 10.1038/s41423-021-00790-w PMC8632917 34728795

[117] Ni X Wang Q Gu J Lu L. Clinical and basic research progress on treg-induced immune tolerance in liver transplantation. Front Immunol (2021) 12:535012. doi: 10.3389/fimmu.2021.535012 34093514 PMC8173171

[118] Zhang H Jiang Z Zhang L. Dual effect of T helper cell 17 (Th17) and regulatory T cell (Treg) in liver pathological process: From occurrence to end stage of disease. Int Immunopharmacol (2019) 69:50–9. doi: 10.1016/j.intimp.2019.01.005 30669025

[119] Osei-Bordom D Bozward AG Oo YH. The hepatic microenvironment and regulatory T cells. Cell Immunol (2020) 357:104195. doi: 10.1016/j.cellimm.2020.104195 32861844

[120] Maria A English KA Gorham JD. Appropriate development of the liver treg compartment is modulated by the microbiota and requires TGF-beta and MyD88. J Immunol Res (2014) 2014:279736. doi: 10.1155/2014/279736 25177709 PMC4142300

[121] Li M Zhao W Wang Y Jin L Jin G Sun X. A wave of Foxp3(+) regulatory T cell accumulation in the neonatal liver plays unique roles in maintaining self-tolerance. Cell Mol Immunol (2020) 17:507–18. doi: 10.1038/s41423-019-0246-9 PMC7193579 31171863

[122] Breous E Somanathan S Vandenberghe LH Wilson JM. Hepatic regulatory T cells and kupffer cells are crucial mediators of systemic T cell tolerance to antigens targeting murine liver. Hepatology (2009) 50:612–21. doi: 10.1002/hep.23043 PMC4380144 19575456

[123] Dywicki J Buitrago-Molina LE Noyan F Davalos-Misslitz AC Hupa-Breier KL Lieber M. The detrimental role of regulatory T cells in nonalcoholic steatohepatitis. Hepatol Commun (2022) 6:320–33. doi: 10.1002/hep4.1807 PMC8793993 34532981

[124] Van Herck MA Vonghia L Kwanten WJ Vanwolleghem T Ebo DG Michielsen PP. Adoptive cell transfer of regulatory T cells exacerbates hepatic steatosis in high-fat high-fructose diet-fed mice. Front Immunol (2020) 11:1711. doi: 10.3389/fimmu.2020.01711 32849604 PMC7412973

[125] Roh YS Kim JW Park S Shon C Kim S Eo SK. Toll-like receptor-7 signaling promotes nonalcoholic steatohepatitis by inhibiting regulatory T cells in mice. Am J Pathol (2018) 188:2574–88. doi: 10.1016/j.ajpath.2018.07.011 30125542

[126] Ikeno Y Ohara D Takeuchi Y Watanabe H Kondoh G Taura K. Foxp3+ regulatory T cells inhibit CCl4-induced liver inflammation and fibrosis by regulating tissue cellular immunity. Front Immunol (2020) 11:584048. doi: 10.3389/fimmu.2020.584048 33178216 PMC7593684

[127] Wang P Shi B Wang C Wang Y Que W Jiang Z. Hepatic pannexin-1 mediates ST2(+) regulatory T cells promoting resolution of inflammation in lipopolysaccharide-induced endotoxemia. Clin Transl Med (2022) 12:e849. doi: 10.1002/ctm2.849 35593197 PMC9121315

[128] Ascon DB Ascon M Satpute S Lopez-Briones S Racusen L Colvin RB. Normal mouse kidneys contain activated and CD3+CD4- CD8- double-negative T lymphocytes with a distinct TCR repertoire. J Leukoc Biol (2008) 84:1400–9. doi: 10.1189/jlb.0907651 PMC2614602 18765477

[129] Pollow DP Jr. Uhlorn JA Sylvester MA Romero-Aleshire MJ Uhrlaub JL Lindsey ML. Menopause and FOXP3(+) treg cell depletion eliminate female protection against T cell-mediated angiotensin II hypertension. Am J Physiol Heart Circ Physiol (2019) 317:H415–H23. doi: 10.1152/ajpheart.00792.2018 PMC6732479 31099612

[130] Xu J Li X Yuan Q Wang C Xu L Wei X. The semaphorin 4A-neuropilin 1 axis alleviates kidney ischemia reperfusion injury by promoting the stability and function of regulatory T cells. Kidney Int (2021) 100:1268–81. doi: 10.1016/j.kint.2021.08.023 34534552

[131] Yang YH Istomine R Alvarez F Al-Aubodah TA Shi XQ Takano T. Salt sensing by Serum/Glucocorticoid-regulated kinase 1 promotes Th17-like inflammatory adaptation of Foxp3(+) regulatory T cells. Cell Rep (2020) 30:1515–29.e4. doi: 10.1016/j.celrep.2020.01.002 32023466 PMC11056843

[132] Belanger KM Crislip GR Gillis EE Abdelbary M Musall JB Mohamed R. Greater T regulatory cells in females attenuate DOCA-Salt-Induced increases in blood pressure versus males. Hypertension (2020) 75:1615–23. doi: 10.1161/HYPERTENSIONAHA.119.14089 PMC7225054 32336228

[133] do Valle Duraes F Lafont A Beibel M Martin K Darribat K Cuttat R. Immune cell landscaping reveals a protective role for regulatory T cells during kidney injury and fibrosis. JCI Insight (2020) 5(3):e130651. doi: 10.1172/jci.insight.130651 PMC7098794 32051345

[134] Miller RP Tadagavadi RK Ramesh G Reeves WB. Mechanisms of cisplatin nephrotoxicity. Toxins (Basel) (2010) 2:2490–518. doi: 10.3390/toxins2112490 PMC3153174 22069563

[135] Timmers L Pasterkamp G de Hoog VC Arslan F Appelman Y de Kleijn DP. The innate immune response in reperfused myocardium. Cardiovasc Res (2012) 94:276–83. doi: 10.1093/cvr/cvs018 22266751

[136] Stremska ME Jose S Sabapathy V Huang L Bajwa A Kinsey GR. IL233, a novel IL-2 and IL-33 hybrid cytokine, ameliorates renal injury. J Am Soc Nephrol (2017) 28:2681–93. doi: 10.1681/ASN.2016121272 PMC5576940 28539382

[137] Lee H Nho D Chung HS Lee H Shin MK Kim SH. CD4+CD25+ regulatory T cells attenuate cisplatin-induced nephrotoxicity in mice. Kidney Int (2010) 78:1100–9. doi: 10.1038/ki.2010.139 20463654

[138] Wang WW Wang Y Li K Tadagavadi R Friedrichs WE Budatha M. IL-10 from dendritic cells but not from T regulatory cells protects against cisplatin-induced nephrotoxicity. PLoS One (2020) 15:e0238816. doi: 10.1371/journal.pone.0238816 32898157 PMC7478814

[139] Alikhan MA Summers SA Gan PY Chan AJ Khouri MB Ooi JD. Endogenous toll-like receptor 9 regulates AKI by promoting regulatory T cell recruitment. J Am Soc Nephrol (2016) 27:706–14. doi: 10.1681/ASN.2014090927 PMC4769185 26116356

[140] Sakai R Ito M Komai K Iizuka-Koga M Matsuo K Nakayama T. Kidney GATA3(+) regulatory T cells play roles in the convalescence stage after antibody-mediated renal injury. Cell Mol Immunol (2021) 18:1249–61. doi: 10.1038/s41423-020-00547-x PMC8093306 32917984

[141] Kasal DA Barhoumi T Li MW Yamamoto N Zdanovich E Rehman A. T Regulatory lymphocytes prevent aldosterone-induced vascular injury. Hypertension (2012) 59:324–30. doi: 10.1161/HYPERTENSIONAHA.111.181123 22146512

[142] Barhoumi T Kasal DA Li MW Shbat L Laurant P Neves MF. T Regulatory lymphocytes prevent angiotensin II-induced hypertension and vascular injury. Hypertension (2011) 57:469–76. doi: 10.1161/HYPERTENSIONAHA.110.162941 21263125

[143] Gillis EE Belanger K Abdelbary M Mohamed R Sun J Brands MW. Splenectomy increases blood pressure and abolishes sex differences in renal T-regulatory cells in spontaneously hypertensive rats. Clin Sci (Lond) (2021) 135:2329–39. doi: 10.1042/CS20210469 34585239

[144] Zhang YJ Shen L Zhang T Muyayalo KP Luo J Mor G. Immunologic memory in pregnancy: Focusing on memory regulatory T cells. Int J Biol Sci (2022) 18:2406–18. doi: 10.7150/ijbs.70629 PMC8990478 35414772

[145] Schumacher A Brachwitz N Sohr S Engeland K Langwisch S Dolaptchieva M. Human chorionic gonadotropin attracts regulatory T cells into the fetal-maternal interface during early human pregnancy. J Immunol (2009) 182:5488–97. doi: 10.4049/jimmunol.0803177 19380797

[146] Quinn KH Lacoursiere DY Cui L Bui J Parast MM. The unique pathophysiology of early-onset severe preeclampsia: role of decidual T regulatory cells. J Reprod Immunol (2011) 91:76–82. doi: 10.1016/j.jri.2011.05.006 21782252

[147] Salvany-Celades M van der Zwan A Benner M Setrajcic-Dragos V Bougleux Gomes HA Iyer V. Three types of functional regulatory T cells control T cell responses at the human maternal-fetal interface. Cell Rep (2019) 27:2537–47.e5. doi: 10.1016/j.celrep.2019.04.109 31141680

[148] Sasaki Y Sakai M Miyazaki S Higuma S Shiozaki A Saito S. Decidual and peripheral blood CD4+CD25+ regulatory T cells in early pregnancy subjects and spontaneous abortion cases. Mol Hum Reprod (2004) 10:347–53. doi: 10.1093/molehr/gah044 14997000

[149] Yang H Qiu L Chen G Ye Z Lu C Lin Q. Proportional change of CD4+CD25+ regulatory T cells in decidua and peripheral blood in unexplained recurrent spontaneous abortion patients. Fertil Steril (2008) 89:656–61. doi: 10.1016/j.fertnstert.2007.03.037 17543960

[150] Inada K Shima T Nakashima A Aoki K Ito M Saito S. Characterization of regulatory T cells in decidua of miscarriage cases with abnormal or normal fetal chromosomal content. J Reprod Immunol (2013) 97:104–11. doi: 10.1016/j.jri.2012.12.001 23432877

[151] Inada K Shima T Ito M Ushijima A Saito S. Helios-Positive functional regulatory T cells are decreased in decidua of miscarriage cases with normal fetal chromosomal content. J Reprod Immunol (2015) 107:10–9. doi: 10.1016/j.jri.2014.09.053 25453751

[152] Jasper MJ Tremellen KP Robertson SA. Primary unexplained infertility is associated with reduced expression of the T-regulatory cell transcription factor Foxp3 in endometrial tissue. Mol Hum Reprod (2006) 12:301–8. doi: 10.1093/molehr/gal032 16574699

[153] Giudice LC Kao LC. Endometriosis. Lancet (2004) 364:1789–99. doi: 10.1016/S0140-6736(04)17403-5 15541453

[154] Berbic M Hey-Cunningham AJ Ng C Tokushige N Ganewatta S Markham R. The role of Foxp3+ regulatory T-cells in endometriosis: a potential controlling mechanism for a complex, chronic immunological condition. Hum Reprod (2010) 25:900–7. doi: 10.1093/humrep/deq020 20150173

[155] Basta P Majka M Jozwicki W Lukaszewska E Knafel A Grabiec M. The frequency of CD25+CD4+ and FOXP3+ regulatory T cells in ectopic endometrium and ectopic decidua. Reprod Biol Endocrinol (2010) 8:116. doi: 10.1186/1477-7827-8-116 20923543 PMC2958978

[156] Takamura M Koga K Izumi G Hirata T Harada M Hirota Y. Simultaneous detection and evaluation of four subsets of CD4+ T lymphocyte in lesions and peripheral blood in endometriosis. Am J Reprod Immunol (2015) 74:480–6. doi: 10.1111/aji.12426 26387982

[157] Chen S Zhang J Huang C Lu W Liang Y Wan X. Expression of the T regulatory cell transcription factor FoxP3 in peri-implantation phase endometrium in infertile women with endometriosis. Reprod Biol Endocrinol (2012) 10:34. doi: 10.1186/1477-7827-10-34 22541024 PMC3443024

[158] Podgaec S Barbeiro DF Gueuvoghlanian-Silva BY Bellelis P Abrao MS Baracat EC. Foxp3 expression in deep rectosigmoid endometriosis lesions and its association with chronic pelvic pain. J Reprod Immunol (2014) 104-105:96–9. doi: 10.1016/j.jri.2014.05.002 25064223

[159] Tanaka Y Mori T Ito F Koshiba A Takaoka O Kataoka H. Exacerbation of endometriosis due to regulatory T-cell dysfunction. J Clin Endocrinol Metab (2017) 102:3206–17. doi: 10.1210/jc.2017-00052 28575420

[160] Xiao F Liu X Guo SW. Platelets and regulatory T cells may induce a type 2 immunity that is conducive to the progression and fibrogenesis of endometriosis. Front Immunol (2020) 11:610963. doi: 10.3389/fimmu.2020.610963 33381124 PMC7767909

[161] Ssemaganda A Cholette F Perner M Kambaran C Adhiambo W Wambugu PM. Endocervical regulatory T cells are associated with decreased genital inflammation and lower HIV target cell abundance. Front Immunol (2021) 12:726472. doi: 10.3389/fimmu.2021.726472 34630402 PMC8495419

[162] Betts CB Pennock ND Caruso BP Ruffell B Borges VF Schedin P. Mucosal immunity in the female murine mammary gland. J Immunol (2018) 201:734–46. doi: 10.4049/jimmunol.1800023 PMC6036228 29884705

[163] Nasri F Doroudchi M Namavar Jahromi B Gharesi-Fard B. T Helper cells profile and CD4+CD25+Foxp3+Regulatory T cells in polycystic ovary syndrome. Iran J Immunol (2018) 15:175–85. doi: 10.22034/IJI.2018.39387 30246693

[164] Liu D Tu X Huang C Yuan Y Wang Y Liu X. Adoptive transfers of CD4(+) CD25(+) tregs partially alleviate mouse premature ovarian insufficiency. Mol Reprod Dev (2020) 87:887–98. doi: 10.1002/mrd.23404 32741069

[165] Wheeler K Tardif S Rival C Luu B Bui E Del Rio R. Regulatory T cells control tolerogenic versus autoimmune response to sperm in vasectomy. Proc Natl Acad Sci USA (2011) 108:7511–6. doi: 10.1073/pnas.1017615108 PMC3088630 21502500

[166] Nicolas N Michel V Bhushan S Wahle E Hayward S Ludlow H. Testicular activin and follistatin levels are elevated during the course of experimental autoimmune epididymo-orchitis in mice. Sci Rep (2017) 7:42391. doi: 10.1038/srep42391 28205525 PMC5304336

[167] Jacobo P. The role of regulatory T cells in autoimmune orchitis. Andrologia (2018) 50:e13092. doi: 10.1111/and.13092 30569653

[168] Jacobo P Guazzone VA Jarazo-Dietrich S Theas MS Lustig L. Differential changes in CD4+ and CD8+ effector and regulatory T lymphocyte subsets in the testis of rats undergoing autoimmune orchitis. J Reprod Immunol (2009) 81:44–54. doi: 10.1016/j.jri.2009.04.005 19520436

[169] Jacobo P Guazzone VA Perez CV Lustig L. CD4+ Foxp3+ regulatory T cells in autoimmune orchitis: phenotypic and functional characterization. Am J Reprod Immunol (2015) 73:109–25. doi: 10.1111/aji.12312 25164316

[170] Tung KS Harakal J Qiao H Rival C Li JC Paul AG. Egress of sperm autoantigen from seminiferous tubules maintains systemic tolerance. J Clin Invest (2017) 127:1046–60. doi: 10.1172/JCI89927 PMC5330742 28218625

[171] Tanoue T Atarashi K Honda K. Development and maintenance of intestinal regulatory T cells. Nat Rev Immunol (2016) 16:295–309. doi: 10.1038/nri.2016.36 27087661

[172] Sharma A Rudra D. Emerging functions of regulatory T cells in tissue homeostasis. Front Immunol (2018) 9:883. doi: 10.3389/fimmu.2018.00883 29887862 PMC5989423

[173] Lui PP Cho I Ali N. Tissue regulatory T cells. Immunology (2020) 161:4–17. doi: 10.1111/imm.13208 32463116 PMC7450170

[174] Shao Q Gu J Zhou J Wang Q Li X Deng Z. Tissue tregs and maintenance of tissue homeostasis. Front Cell Dev Biol (2021) 9:717903. doi: 10.3389/fcell.2021.717903 34490267 PMC8418123

[175] Munoz-Rojas AR Mathis D. Tissue regulatory T cells: regulatory chameleons. Nat Rev Immunol (2021) 21:597–611. doi: 10.1038/s41577-021-00519-w 33772242 PMC8403160

[176] Traxinger BR Richert-Spuhler LE Lund JM. Mucosal tissue regulatory T cells are integral in balancing immunity and tolerance at portals of antigen entry. Mucosal Immunol (2022) 15:398–407. doi: 10.1038/s41385-021-00471-x 34845322 PMC8628059

[177] Estrada Brull A Panetti C Joller N. Moving to the outskirts: Interplay between regulatory T cells and peripheral tissues. Front Immunol (2022) 13:864628. doi: 10.3389/fimmu.2022.864628 35572535 PMC9099010

[178] Wohlfert EA Grainger JR Bouladoux N Konkel JE Oldenhove G Ribeiro CH. GATA3 controls Foxp3(+) regulatory T cell fate during inflammation in mice. J Clin Invest (2011) 121:4503–15. doi: 10.1172/JCI57456 PMC3204837 21965331

[179] Sefik E Geva-Zatorsky N Oh S Konnikova L Zemmour D McGuire AM. Mucosal immunology. individual intestinal symbionts induce a distinct population of RORgamma(+) regulatory T cells. Science (2015) 349:993–7. doi: 10.1126/science.aaa9420 PMC4700932 26272906

[180] Neumann C Blume J Roy U Teh PP Vasanthakumar A Beller A. C-maf-dependent treg cell control of intestinal TH17 cells and IgA establishes host-microbiota homeostasis. Nat Immunol (2019) 20:471–81. doi: 10.1038/s41590-019-0316-2 30778241

[181] Xu M Pokrovskii M Ding Y Yi R Au C Harrison OJ. C-MAF-dependent regulatory T cells mediate immunological tolerance to a gut pathobiont. Nature (2018) 554:373–7. doi: 10.1038/nature25500 PMC5814346 29414937

[182] Ohnmacht C Park JH Cording S Wing JB Atarashi K Obata Y. Mucosal immunology. the microbiota regulates type 2 immunity through RORgammat(+) T cells. Science (2015) 349:989–93. doi: 10.1126/science.aac4263 26160380

[183] Kim KS Hong SW Han D Yi J Jung J Yang BG. Dietary antigens limit mucosal immunity by inducing regulatory T cells in the small intestine. Science (2016) 351:858–63. doi: 10.1126/science.aac5560 26822607

[184] Atarashi K Tanoue T Shima T Imaoka A Kuwahara T Momose Y. Induction of colonic regulatory T cells by indigenous clostridium species. Science (2011) 331:337–41. doi: 10.1126/science.1198469 PMC3969237 21205640

[185] Schiering C Krausgruber T Chomka A Frohlich A Adelmann K Wohlfert EA. The alarmin IL-33 promotes regulatory T-cell function in the intestine. Nature (2014) 513:564–8. doi: 10.1038/nature13577 PMC4339042 25043027

[186] Krzyzanowska AK Haynes Ii RAH Kovalovsky D Lin HC Osorio L Edelblum KL. Zbtb20 identifies and controls a thymus-derived population of regulatory T cells that play a role in intestinal homeostasis. Sci Immunol (2022) 7:eabf3717. doi: 10.1126/sciimmunol.abf3717 35522722 PMC9709958

[187] Miragaia RJ Gomes T Chomka A Jardine L Riedel A Hegazy AN. Single-cell transcriptomics of regulatory T cells reveals trajectories of tissue adaptation. Immunity (2019) 50:493–504.e7. doi: 10.1016/j.immuni.2019.01.001 30737144 PMC6382439

[188] Scharschmidt TC Vasquez KS Truong HA Gearty SV Pauli ML Nosbaum A. A wave of regulatory T cells into neonatal skin mediates tolerance to commensal microbes. Immunity (2015) 43:1011–21. doi: 10.1016/j.immuni.2015.10.016 PMC4654993 26588783

[189] Kalekar LA Cohen JN Prevel N Sandoval PM Mathur AN Moreau JM. Regulatory T cells in skin are uniquely poised to suppress profibrotic immune responses. Sci Immunol (2019) 4(39):eaaw2910. doi: 10.1126/sciimmunol.aaw2910 31492709 PMC6848056

[190] Nosbaum A Prevel N Truong HA Mehta P Ettinger M Scharschmidt TC. Cutting edge: Regulatory T cells facilitate cutaneous wound healing. J Immunol (2016) 196:2010–4. doi: 10.4049/jimmunol.1502139 PMC4761457 26826250

[191] Ali N Zirak B Rodriguez RS Pauli ML Truong HA Lai K. Regulatory T cells in skin facilitate epithelial stem cell differentiation. Cell (2017) 169:1119–29.e11. doi: 10.1016/j.cell.2017.05.002 28552347 PMC5504703

[192] Alvarez F Istomine R Shourian M Pavey N Al-Aubodah TA Qureshi S. The alarmins IL-1 and IL-33 differentially regulate the functional specialisation of Foxp3(+) regulatory T cells during mucosal inflammation. Mucosal Immunol (2019) 12:746–60. doi: 10.1038/s41385-019-0153-5 30872761

[193] Kanno T Nakajima T Kawashima Y Yokoyama S Asou HK Sasamoto S. Acsbg1-dependent mitochondrial fitness is a metabolic checkpoint for tissue treg cell homeostasis. Cell Rep (2021) 37:109921. doi: 10.1016/j.celrep.2021.109921 34758300

[194] Michalek RD Gerriets VA Jacobs SR Macintyre AN MacIver NJ Mason EF. Cutting edge: distinct glycolytic and lipid oxidative metabolic programs are essential for effector and regulatory CD4+ T cell subsets. J Immunol (2011) 186:3299–303. doi: 10.4049/jimmunol.1003613 PMC3198034 21317389

[195] Raud B Roy DG Divakaruni AS Tarasenko TN Franke R Ma EH. Etomoxir actions on regulatory and memory T cells are independent of Cpt1a-mediated fatty acid oxidation. Cell Metab (2018) 28:504–15.e7. doi: 10.1016/j.cmet.2018.06.002 30043753 PMC6747686

[196] Hou J Sun Y Hao Y Zhuo J Liu X Bai P. Imbalance between subpopulations of regulatory T cells in COPD. Thorax (2013) 68:1131–9. doi: 10.1136/thoraxjnl-2012-201956 23749814

[197] Sales DS Ito JT Zanchetta IA Annoni R Aun MV Ferraz LFS. Regulatory T-cell distribution within lung compartments in COPD. COPD (2017) 14:533–42. doi: 10.1080/15412555.2017.1346069 28745532

[198] Eriksson Strom J Pourazar J Linder R Blomberg A Lindberg A Bucht A. Airway regulatory T cells are decreased in COPD with a rapid decline in lung function. Respir Res (2020) 21:330. doi: 10.1186/s12931-020-01593-9 33317530 PMC7734742

[199] Cervilha DAB Ito JT Lourenco JD Olivo CR Saraiva-Romanholo BM Volpini RA. The Th17/Treg cytokine imbalance in chronic obstructive pulmonary disease exacerbation in an animal model of cigarette smoke exposure and lipopolysaccharide challenge association. Sci Rep (2019) 9:1921. doi: 10.1038/s41598-019-38600-z 30760822 PMC6374436

[200] Ito JT Cervilha DAB Lourenco JD Goncalves NG Volpini RA Caldini EG. Th17/Treg imbalance in COPD progression: A temporal analysis using a CS-induced model. PLoS One (2019) 14:e0209351. doi: 10.1371/journal.pone.0209351 30629626 PMC6328193

[201] Silva LEF Lourenco JD Silva KR Santana FPR Kohler JB Moreira AR. Th17/Treg imbalance in COPD development: suppressors of cytokine signaling and signal transducers and activators of transcription proteins. Sci Rep (2020) 10:15287. doi: 10.1038/s41598-020-72305-y 32943702 PMC7499180

[202] D’Alessio FR Tsushima K Aggarwal NR West EE Willett MH Britos MF. CD4+CD25+Foxp3+ tregs resolve experimental lung injury in mice and are present in humans with acute lung injury. J Clin Invest (2009) 119:2898–913. doi: 10.1172/JCI36498 PMC2752062 19770521

[203] Mock JR Garibaldi BT Aggarwal NR Jenkins J Limjunyawong N Singer BD. Foxp3+ regulatory T cells promote lung epithelial proliferation. Mucosal Immunol (2014) 7:1440–51. doi: 10.1038/mi.2014.33 PMC4205163 24850425

[204] Dial CF Tune MK Doerschuk CM Mock JR. Foxp3(+) regulatory T cell expression of keratinocyte growth factor enhances lung epithelial proliferation. Am J Respir Cell Mol Biol (2017) 57:162–73. doi: 10.1165/rcmb.2017-0019OC PMC5576587 28296468

[205] Arpaia N Green JA Moltedo B Arvey A Hemmers S Yuan S. A distinct function of regulatory T cells in tissue protection. Cell (2015) 162:1078–89. doi: 10.1016/j.cell.2015.08.021 PMC4603556 26317471

[206] Liu Q Dwyer GK Zhao Y Li H Mathews LR Chakka AB. IL-33-mediated IL-13 secretion by ST2+ tregs controls inflammation after lung injury. JCI Insight (2019) 4(6):e123919. doi: 10.1172/jci.insight.123919 PMC6482994 30779711

[207] Ehrentraut H Clambey ET McNamee EN Brodsky KS Ehrentraut SF Poth JM. CD73+ regulatory T cells contribute to adenosine-mediated resolution of acute lung injury. FASEB J (2013) 27:2207–19. doi: 10.1096/fj.12-225201 PMC3659359 23413361

[208] Mock JR Dial CF Tune MK Norton DL Martin JR Gomez JC. Transcriptional analysis of Foxp3+ tregs and functions of two identified molecules during resolution of ALI. JCI Insight (2019) 4(6):e124958. doi: 10.1172/jci.insight.124958 PMC6482998 30753170

[209] Lo Re S Lecocq M Uwambayinema F Yakoub Y Delos M Demoulin JB. Platelet-derived growth factor-producing CD4+ Foxp3+ regulatory T lymphocytes promote lung fibrosis. Am J Respir Crit Care Med (2011) 184:1270–81. doi: 10.1164/rccm.201103-0516OC 21868503

[210] Garibaldi BT D’Alessio FR Mock JR Files DC Chau E Eto Y. Regulatory T cells reduce acute lung injury fibroproliferation by decreasing fibrocyte recruitment. Am J Respir Cell Mol Biol (2013) 48:35–43. doi: 10.1165/rcmb.2012-0198OC 23002097 PMC3547087

[211] Mohseni YR Tung SL Dudreuilh C Lechler RI Fruhwirth GO Lombardi G. The future of regulatory T cell therapy: Promises and challenges of implementing CAR technology. Front Immunol (2020) 11:1608. doi: 10.3389/fimmu.2020.01608 32793236 PMC7393941

